# Molecular tracking of insulin resistance and inflammation development on visceral adipose tissue

**DOI:** 10.3389/fimmu.2023.1014778

**Published:** 2023-03-21

**Authors:** Antonio Bensussen, José Antonio Torres-Magallanes, Elena Roces de Álvarez-Buylla

**Affiliations:** Laboratorio de Neuroendocrinología, Centro Universitario de Investigaciones Biomédicas, Universidad de Colima, Colima, Mexico

**Keywords:** visceral adipose tissue, CD4+ T cells, macrophages, adipocytes, insulin resistance, diabetes mellitus, stochastic dynamic network models

## Abstract

**Background:**

Visceral adipose tissue (VAT) is one of the most important sources of proinflammatory molecules in obese people and it conditions the appearance of insulin resistance and diabetes. Thus, understanding the synergies between adipocytes and VAT-resident immune cells is essential for the treatment of insulin resistance and diabetes.

**Methods:**

We collected information available on databases and specialized literature to construct regulatory networks of VAT resident cells, such as adipocytes, CD4+ T lymphocytes and macrophages. These networks were used to build stochastic models based on Markov chains to visualize phenotypic changes on VAT resident cells under several physiological contexts, including obesity and diabetes mellitus.

**Results:**

Stochastic models showed that in lean people, insulin produces inflammation in adipocytes as a homeostatic mechanism to downregulate glucose intake. However, when the VAT tolerance to inflammation is exceeded, adipocytes lose insulin sensitivity according to severity of the inflammatory condition. Molecularly, insulin resistance is initiated by inflammatory pathways and sustained by intracellular ceramide signaling. Furthermore, our data show that insulin resistance potentiates the effector response of immune cells, which suggests its role in the mechanism of nutrient redirection. Finally, our models show that insulin resistance cannot be inhibited by anti-inflammatory therapies alone.

**Conclusion:**

Insulin resistance controls adipocyte glucose intake under homeostatic conditions. However, metabolic alterations such as obesity, enhances insulin resistance in adipocytes, redirecting nutrients to immune cells, permanently sustaining local inflammation in the VAT.

## Introduction

Insulin resistance is a clinical condition in which various cell types stop responding adequately to this hormone (1). Currently, around 463 million of people around the world suffer from this condition (2), mainly due to obesity, sedentary lifestyle and poor nutritional habits. It is estimated that the incidence of people with insulin resistance will increase over time, and may become an extended public health issue world-wide (3). For this reason, numerous efforts have been made to understand the underlying molecular mechanisms of insulin resistance, and how to prevent or revert this pathological condition. It is now known that insulin resistance is has an inflammatory origin and it has been reported that once insulin resistance is generated in the visceral adipose tissue (4) (VAT), this pathological condition can be become systemic. Regarding the causes of insulin resistance in the VAT, some studies have suggested that diets rich in fat and sugar promote the swelling of adipocytes (5), which become inflamed and promote the infiltration of macrophages into the VAT (6). Consequently, localized inflammation is triggered in the VAT, which contributes to promoting obesity and insulin resistance (7). Nevertheless, the exact mechanism by which insulin resistance is generated in adipocytes remains to be elucidated.

To delve into the origin of insulin resistance and understand what are the differential factors that determine the irreversibility of this pathological condition in diabetics, new integrative and innovative approaches such as transcriptomics have been used. The transcriptomic assays performed on diabetic patients showed a strong increase in the activity of the immune system, particularly on CD4+ T lymphocytes and macrophages, coupled to metabolic alterations on adipocytes such as reduction on PPARγ, GLUT4 and adiponectin levels (8). Concerning the macrophages, a significant increase in M1 phenotype on diabetic patients compared to healthy subjects has been observed (9). Regarding CD4+ T cells, recent evidence suggest that Th2 population present a significant reduction while the Th1 and Th17 populations increase in diabetic patients (10). Interestingly, it has been reported, that diabetic patients treated with insulin present a significant increase in IL-10 producing CD4+ T cells (11). These facts are relevant to understand the *in vivo* dynamics of VAT, although, it would be more enriching to have a mechanism that explains how these separate observations are originated at the molecular level. Nonetheless, studying the VAT dynamics *in situ* can be a highly complex task. For this reason, different computational tools have been developed in order to integrate VAT available information and propose new hypotheses that allow an in-depth understanding of how this tissue is deregulated under metabolic diseases such as diabetes.

**Figure 1:**
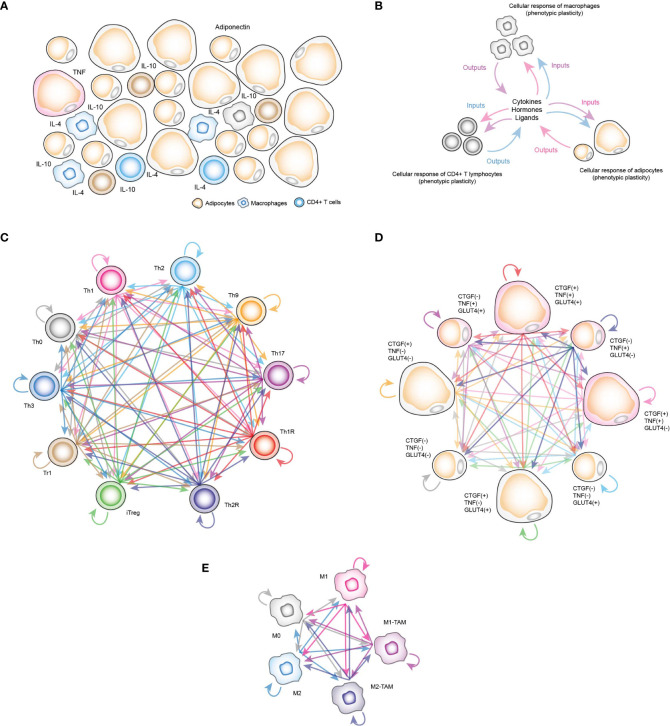
Computational stochastic models to represent the VAT. **(A)** Schematic representation of the VAT with adipocytes, resident macrophages and CD4+ T lymphocytes. **(B)** Schematic representation of functioning VAT model. **(C)** Stochastic model of all phenotypes of CD4+ T cells considered in this work. Each arrow represents a stochastic transition between each phenotype, and the probability of transition between states is determined by the microenvironment signals present in the VAT. **(D)** Stochastic model of phenotypes of adipocytes. Each phenotype represents determined metabolic and genetic state of these cells in response to insulin, and other chemical components of VAT. **(E)** Stochastic model of macrophage phenotypes.

Currently, computational models have been used to study some of the associated effects of insulin resistance on some of the constituent cells of VAT, such as CD4+ T lymphocytes. Specifically, simulations using a gene regulation network (GRN) that models lymphocyte differentiation and plasticity and cell fate under different stimuli (12), was able to predict that hyperinsulinemia tends to polarize lymphocytes towards a Th17 response and at the same time T-regulatory (Treg) cells are reduced (13), which implies that the high levels of insulin present in patients with resistance to this hormone would increase the inflammatory response in VAT. On the other hand, a model based on Ordinary Differential Equations (ODEs) focused on adipocytes showed that adiponectin secretion has an ATP-dependent step to be carried out (14). This finding is important, since adiponectin is a hormone secreted by adipocytes that is responsible for reducing inflammation in VAT (14). Another model of ODEs focused on abdominal subcutaneous adipose tissue was used to estimate the effect of caloric restriction in a group of volunteers and to visualize the metabolic fluxes inside the adipose tissue (15). Nevertheless, it is still necessary to have a computational tool that allows us to visualize the interactions between VAT-resident immune cells with adipocytes in different physiological contexts. In this direction, we constructed a stochastic model based on discrete Markov chains to represent the VAT of healthy, obese and diabetic patients (**Figure 1A**) in order to identify the mechanism by which insulin resistance is generated in adipocytes and to understand how exactly immune cells participate in the appearance of this clinical dysregulation. The model is composed by three sub-models of adipocytes, CD4+ T cells and macrophages. Each sub-model considers chemical components present in the microenvironment of VAT, such as hormones, metabolites and cytokines as inputs to trigger specific responses (**Figure 1B**).

In the case of CD4+ T lymphocytes, the phenotypes considered were Th0 lymphocytes, effector variants Th1, Th2, Th9 and Th17, as well as regulatory phenotypes such as Th1R (FoxP3+ IFN-γ +), Th2R (FoxP3+ IL-4+), iTreg (FoxP3+ IL-10+ TGF-β+), Tr1 (FoxP3- IL-10+) and Th3 (FoxP3- TGF-β+) (**Figure 1C**). For the adipocytes model, we considered the following observations: TNF is expressed only in inflamed adipocytes (16), while cells that express connective tissue growth factor (CTGF) are hypertrophic adipocytes (16), and similarly, adipocytes that translocate GLUT4 (8) are responsive to insulin. Considering these three experimentally tested markers, as well as recent experimental evidence that suggest functional phenotypic diversity of adipocytes (17), we proposed a series of phenotypes in which adipocytes might have combinations of these genes turned on and/or turned off. These eight phenotypes are “TNF- CTGF- GLUT4+”, “TNF- CTGF+ GLUT4-”, “TNF- CTGF+ GLUT4+”, “TNF+ CTGF- GLUT4-”, “TNF+ CTGF- GLUT4+”, “TNF+ CTGF+ GLUT4-”, “TNF+ CTGF+ GLUT4+”, “GLUT4- CTGF- TNF-” (**Figure 1D**). Finally, the macrophage model considers monocytes M0, polarized macrophages M1, M2 and tumor-associated macrophages (TAMs) type M1 (M1-TAM) and type M2 (M2-TAM) (**Figure 1E**). Using this computational approach, we found that insulin naturally creates inflammation in VAT cells as a normal part of the nutrient absorption process, although adipocytes compensate this local inflammation with the production of adiponectin. However, under obesity or diabetes, this balance is broken, generating insulin resistance in adipocytes. Our results showed that the severity of insulin resistance depends on the degree of inflammation present in the tissue. Mechanistically, our data show that insulin resistance is generated when pro-inflammatory cytokines activate ceramide signaling, which supports this process in general. Finally, we discuss the possible physiological role of this mechanism embedded in adipocytes and in other insulin-responsive cells.

## Materials and methods

### Methodology overview

**Figure 2:**
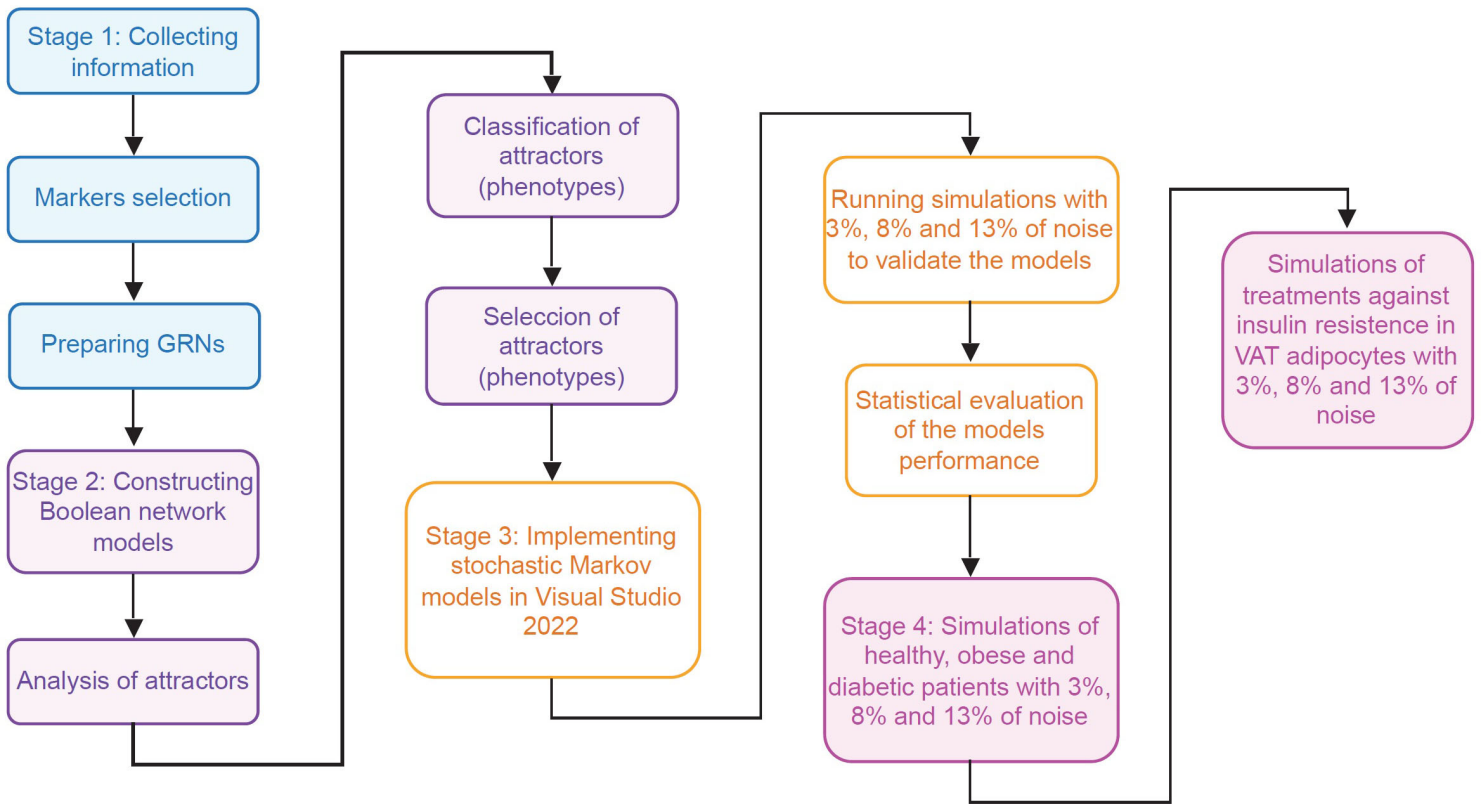
Flowchart of the methodology. This work was carried out in four different stages. In the first stage (blue rectangles) the necessary information was collected to model the main VAT cells. In the second stage (purple rectangles) models of Boolean networks of CD4+ T lymphocytes, macrophages and adipocytes (**Supplementary Information**) were built and evaluated. In the third stage (yellow rectangles), the information of previous stages was used to build and validate stochastic models based on Markov chains of adipocytes, CD4+ T lymphocytes, and VAT-resident macrophages. Finally, in the fourth stage (pink rectangles), exploratory simulations were carried out to study the operation of the VAT in different pathophysiological contexts and its response to possible pharmacological treatments.

In order to track how insulin resistance is generated in VAT adipocytes, we divided this work in four stages. During the first stage, information was collected about the intracellular functioning of CD4+ T lymphocytes, macrophages and adipocytes, considering the particularities of VAT; and for this, we use databases and available specialized literature (**Figure 2**). In the second stage, we use the collected information to create Boolean network models of macrophages and adipocytes. Furthermore, we expanded a model of CD4+ T cells developed by Martinez et al. (12), to visualize the Th9 phenotype. Subsequently, we calculated the attractors of each model, and classified them into phenotypes based on the gene expression pattern they presented (**Figure 2**) (**Supplementary Information**). In the third stage of this work, we build the stochastic models based on Markov chains, and we focus on validating the qualitative behavior of each model (**Figure 2**). In the fourth stage of this work, stochastic simulations of different physiological contexts were carried out. Such contexts are the functioning of the VAT in healthy patients, obese patients and diabetic patients. Similarly, the effect of therapeutic agents on VAT adipocytes to reverse insulin resistance was simulated (**Figure 2**).

### Selection of cell markers

To identify macrophage phenotypes, the following molecular markers were selected: iNOS for M1 macrophages (18), Arg1 for M2 macrophages (18), co-expression of Arg1 and iNOS together with IL-12 or IFN-γ (19) for M1-like TAM macrophages (20), and co-expression of Arg1 and iNOS for M2-like TAM macrophages (21). To identify the different lineages of CD4+ T lymphocytes, the following molecular markers were used: IFN-γ and IL-12 for the Th1 phenotype (22); GATA3 and IL-4 for the Th2 phenotype (23); PU.1 and IL-9 for the Th9 phenotype (24); RORγT and IL-17 for the Th17 phenotype (22), and TGF-β, IL-10 and FoxP3 for the regulatory phenotypes (25). Finally, to identify adipocytes, the following markers were used: CTGF for hypertrophic adipocytes (16), TNF for inflamed adipocytes (16), and GLUT4 in the membrane for insulin-responsive adipocytes (8). These markers were selected from purified cell types. This information was used to classify attractors of the Boolean models (**Supplementary information**).

### Validation of phenotype labelling algorithm

To test the efficacy of our algorithm to classify the cellular phenotypes of Boolean attractors, we first searched in GEO (Gene Expression Omnibus) database for a dataset of phenotypes that were identifiable by specialized bioinformatics tools for immune cell detection, such as xCell software (26). In this case, we use data from purified CD4+ T lymphocytes. These RNA seq data are available under accession number GSE210222 and were obtained by Kanno et al. (27). We normalized the dataset under Transcripts Per Million (TPM) convention, after that we calculated the mean expression for each gene. We use this metric to discretize the data values expressed in TPM as follows: we assign 0 to all values below the mean and 1 to all values greater than or equal to the mean. The data in TPM was analyzed with the xCell R package, and the discretized data was analyzed with our attractor classification algorithm. The results of these analyzes are reported in Data File 1.

### Stochastic modeling

To create the stochastic models used in this work, we consulted the specialized literature to create gene regulation networks (GRN) for macrophages and adipocytes. Next, all GRNs were simplified and we used such reduced networks to propose Boolean models for each network (**Supplementary Information**). In the case of CD4+ T lymphocytes, we used the model previously published by Martinez et al. (12, 13), and we added IL-9 signaling and the regulation of the transcriptional factor PU.1 to represent Th9 phenotype (**Supplementary Information**). Next, we search for the attractors and its basins of attraction for each Boolean model, and we selected the most frequent and representative attractors that represent distinctive genotypic characteristics of each phenotype (**Supplementary Information**). Subsequently, we use the reduced GRN of each cell type together with the attractors that represent the studied phenotypes with the previous selected markers to perform the implementation of three discrete Markov chains.

### Computational implementation

To implement the three Markov chain models, we used the C# object-oriented programming language in Microsoft Visual Studio 2022. Each Markov chain was implemented as follows: 1) Attractors that represent the phenotypes studied were used as initial conditions for simulations (**Supplementary Information**). 2) We assigned a noise level associated for each simulation, for the robustness analysis of the networks, noise levels of 3%, 8% and 13% were chosen. For the rest of the simulations, 8% noise was used. 3) For each gene and each time step a stochastic perturbation was simulated by generating a random number uniformly distributed in the interval of (0, 1). If the number was lower than the noise level, then the Boolean function that controls the state of the node (i.e., gene) will give the complement of the value that it should normally report. 4) For each GRN attractor we use 10000-time steps and 30 iterations per phenotype. Subsequently, we repeated this sequence of experiments 10 times and counted the how many times the attractor used as the initial condition was maintained at the end of each simulation. 5) At the end, we divided the total number of times the attractor was conserved by the total number of jumps recorded between the states belonging to the Markov chain, and we reported these data in Data File 2. 6) Finally, we averaged the values for each of the 10 simulation rounds to obtain an average value of the transition probability between each of the states. With this information we create the Markov matrices associated with each model. All matrices and their corresponding conditions of simulation are available in Data File 2.

### Code availability

The code used for each stochastic model presented in this work is available at the ZENODO repository (28).

### Analysis of Markov matrices

We use the final values of each of the Markov matrices (Data File 2), to implement the following equation in MATLAB version 7.0:


$$\vec{P}\left(t\right)=A^{t}\vec{x}$$

Where $\vec{P}\left(t\right)$ is the vector of probability of each Markov chain at any given time *t*, *A* is the transpose of a Markov matrix, and the vector $\vec{x}$ is a vectorial initial condition. We solve this equation for *t* → ∞ in order to obtain the stationary distribution of probabilities for each Markov chain, which corresponds to the distribution of phenotypes of all cell linages (29).

### Statistics

We used the R software (30) to test the qualitative behavior of all models by performing a Binomial test of one tail, to determine whether the probability of success of each stochastic model was higher than the randomness (p = 0.5) or not. We also used the R software to test the quantitative accuracy of each model by performing a multivariate correlation analysis. In both procedures we used 5% of significance.

### Data availability

**Figure 3:**
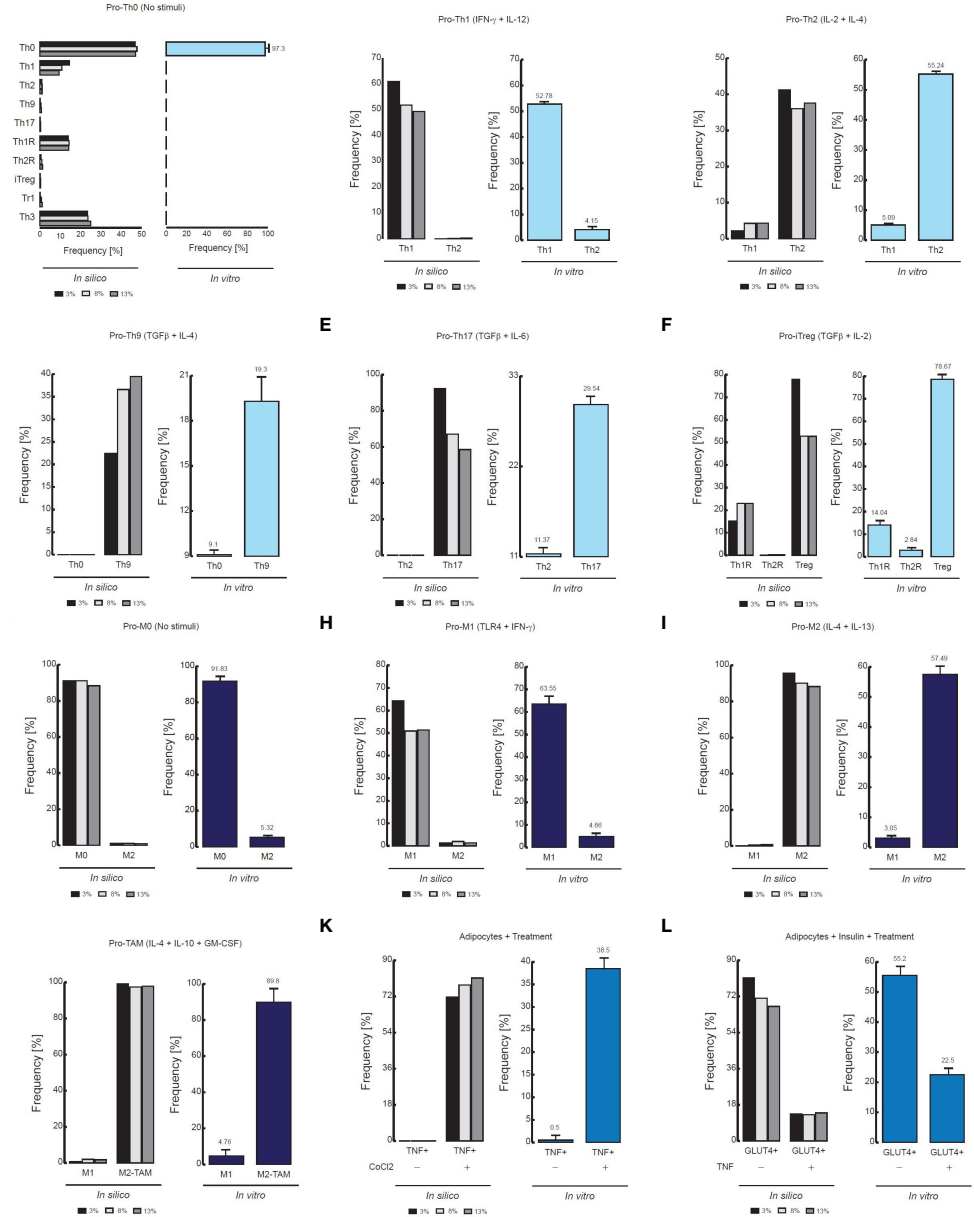
Comparison of flow cytometry data *vs in silico* proportions of cell phenotypes. **(A)** Phenotype distribution of CD4+ T cells without stimuli, **(B)** in presence of IFN-γ and IL-12, **(C)** in presence of IL-2 and IL-4, **(D)** TGF-β and IL-4, **(E)** TGF-β and IL-6, **(F)** TGF−β and IL-2. For panels D, the flow cytometry data was adapted from (24). The data of the remaining panels was adapted from (31). **(G)** Phenotype distribution of Macrophages without stimuli, **(H)** in presence of IFN- γ and TLR4 ligands, **I** in presence of IL-4 and IL-13, **(J)** In presence of GM-CSF, IL-4 and IL-10. The data of **(G–I)** was adapted from (32), and the data of **(J)** was adapted from (33). **(K)** Phenotypic distribution of adipocytes in presence of an inflammation inducer, **(L)** in presence of TNF. For both panels the data was adapted from (34).

**Figure 4:**
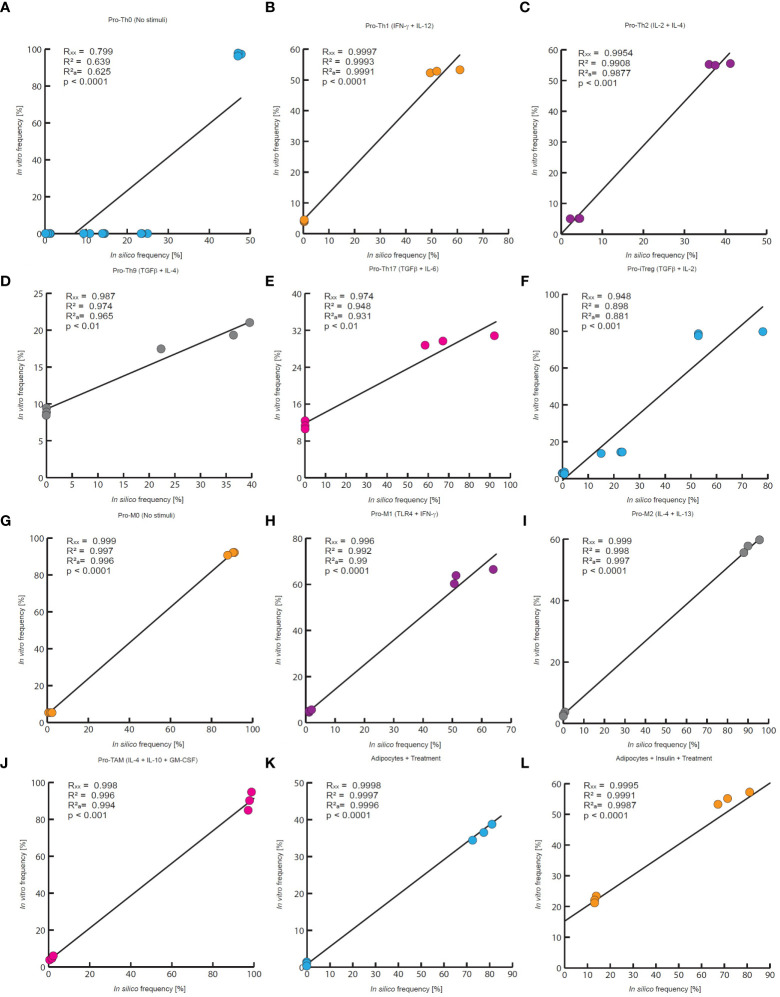
Quantitative evaluation of stochastic models of the VAT cells. In this work the quantitative accuracy of all models was tested by a multivariate correlation analysis. In all panels are compared the outcomes of each model to experimental measurements of every phenotype frequency. Each panel reports the multiple correlation coefficient (R_xx_), the Pearson correlation coefficient (R^2^), the adjusted correlation coefficient ( ${\text{R}}_{a}^{2}$ ) as well as the p-value. **(A)** *in silico* outcomes vs *in vitro* data of CD4+ T cells without stimuli, **(B)** CD4+ T cells treated with IFN-γ and IL-12, **(C)** IL-2 and IL-4, **(D)** TGF-β and IL-4, **(E)** TGF-β and IL-6, **(F)** TGF−β and IL-2. The data for **(A–C, E)**, and **(F)** was taken from (31), and the data for panel D was obtained from (24). **(G)** Macrophages without treatment, **(H)** macrophages treated with IFN- γ and TLR4 ligands, **(I)** IL-4 and IL-13, **(J)** GM-CSF, IL-4 and IL-10. The experimental data of **(G–I)** was taken from (32), and the data of panel J was taken from (33). **(K)** Adipocytes treated with an inflammation inducer, **(L)** and adipocytes treated with TNF. For both panels the experimental data was taken from (34).

**Figure 5:**
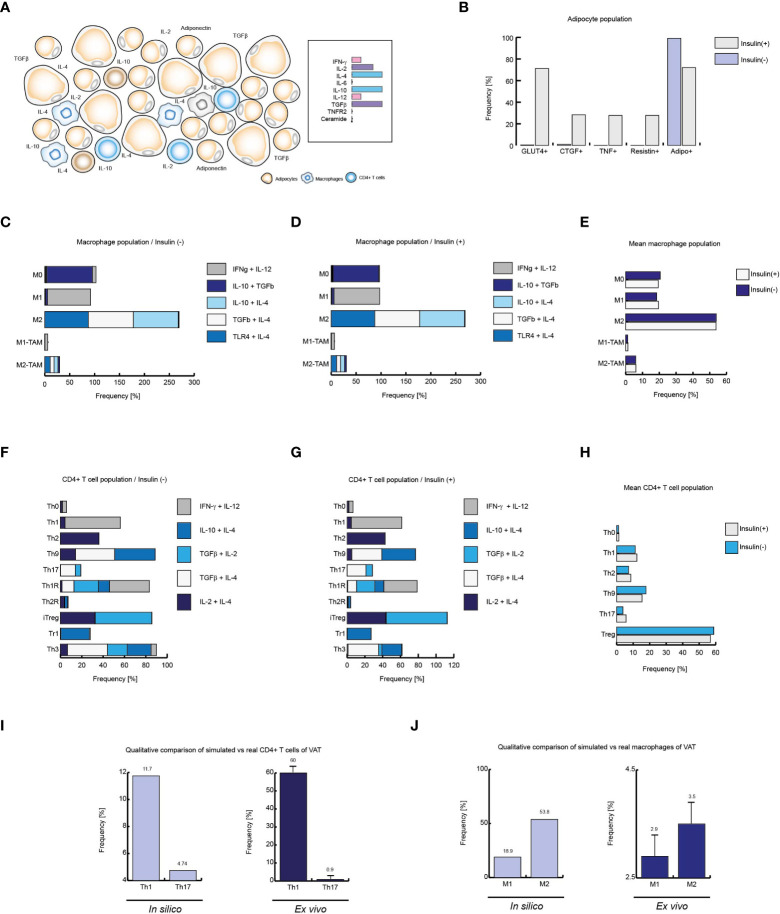
Insulin produces local inflammation on VAT cells. **(A)** Schematic representation of the simulated conditions within healthy lean subjects VAT, **(B)** Cell distribution of adipocytes with and without insulin. **(C)** Distribution of macrophage phenotypes without insulin, and **D**: with insulin. **(E)** Mean behavior of both phenotypic distributions. **(F)** Phenotype distribution of CD4+ T cells in absence of insulin, **(G)** and in presence of high levels of insulin. **(H)** Mean distribution of CD4+ T lymphocytes phenotypes. **(I)** Simulated Th1 and Th17 populations *versus* real frequencies of such phenotypes. **(J)** Simulated M1 and M2 populations *versus* their *ex vivo* frequencies. The values presented for panels **(I, J)** was adapted from (35) and (9) respectively. Collectively, these data suggest that insulin promotes inflammation in healthy VAT of lean people.

**Figure 6:**
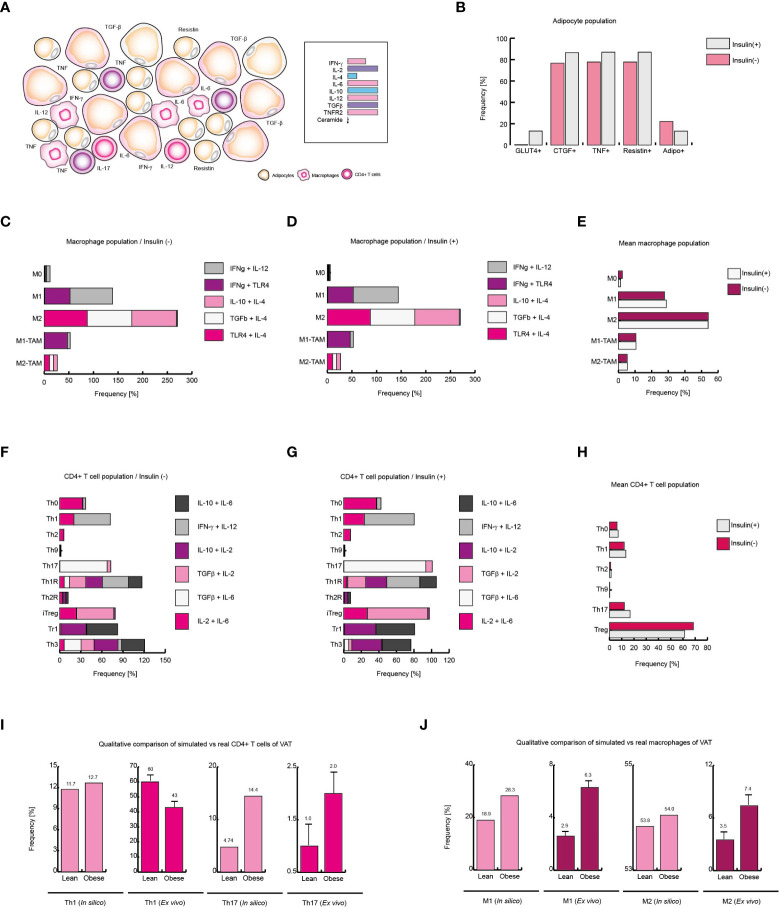
Obesity increases local Th17 immunity in VAT. **(A)** Schematic representation of the simulated conditions within VAT of obese patients. **(B)** Phenotype distribution of adipocytes with and without insulin. **(C)** Phenotype distribution of macrophages in absence of insulin, and **(D)** in presence of insulin. **(E)** Mean behavior of macrophage phenotype distribution. **(F)** Distribution of CD4+ T cells without insulin, **(G)** and with insulin. **(H)** Mean distribution of CD4+ T lymphocytes phenotypes. **(I)** Simulated Th1 and Th17 populations *versus* real frequencies of such phenotypes. **(J)** Simulated M1 and M2 populations *versus* their *ex vivo* frequencies. The values presented for panels **(I, J)** was adapted from (35) and (9) respectively. Collectively, these data suggest that insulin enhances Th17 immunity in obese patients.

**Figure 7:**
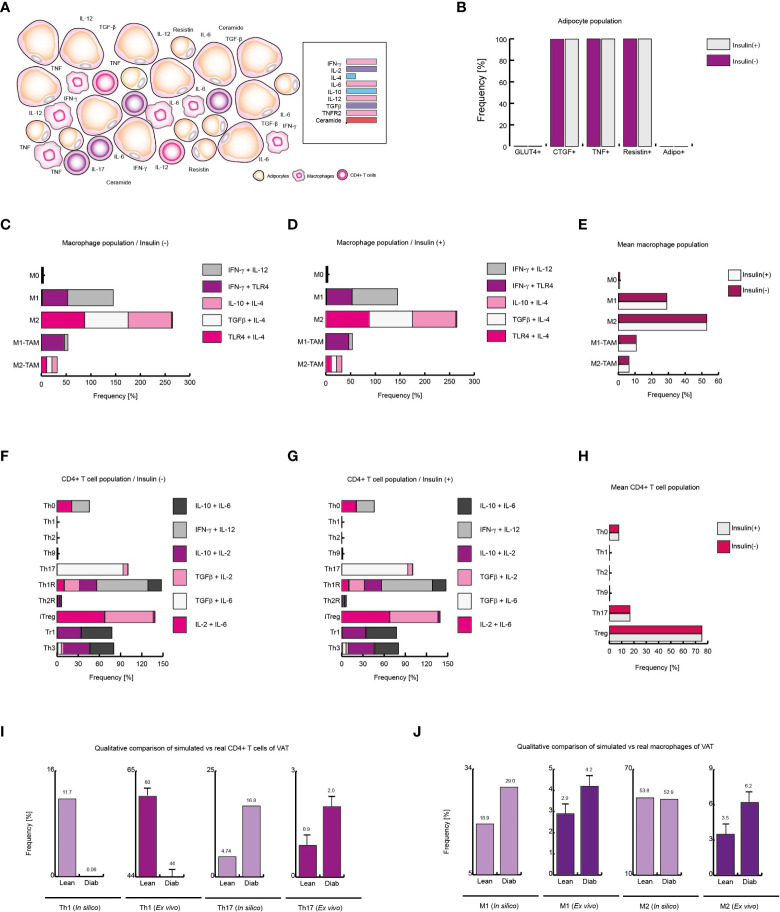
Extracellular ceramide inhibits Th1 response in diabetic patients. **(A)** Schematic representation of the simulated conditions within diabetic patients VAT, **(B)** Distribution of adipocyte-phenotypes with and without insulin. **(C)** Distribution of macrophage-phenotypes in absence of insulin, and **(D)** in presence of insulin. **(E)** Average behavior of macrophage phenotype distribution. **(F)** Distribution of CD4+ T cells without insulin, **(G)** and with insulin. **(H)** Average distribution of CD4+ T lymphocytes phenotypes. **(I)** Simulated Th1 and Th17 populations compared to *ex vivo* frequencies of such phenotypes. **(J)** Simulated M1 and M2 populations *versus* their *ex vivo* frequencies. The values presented for panels **(I, J)** was adapted from (35) and (9) respectively. These results suggest that extracellular ceramide inhibits Th1 response and avoids Treg inhibition due to insulin, as it was observed in obese patients.

**Figure 8:**
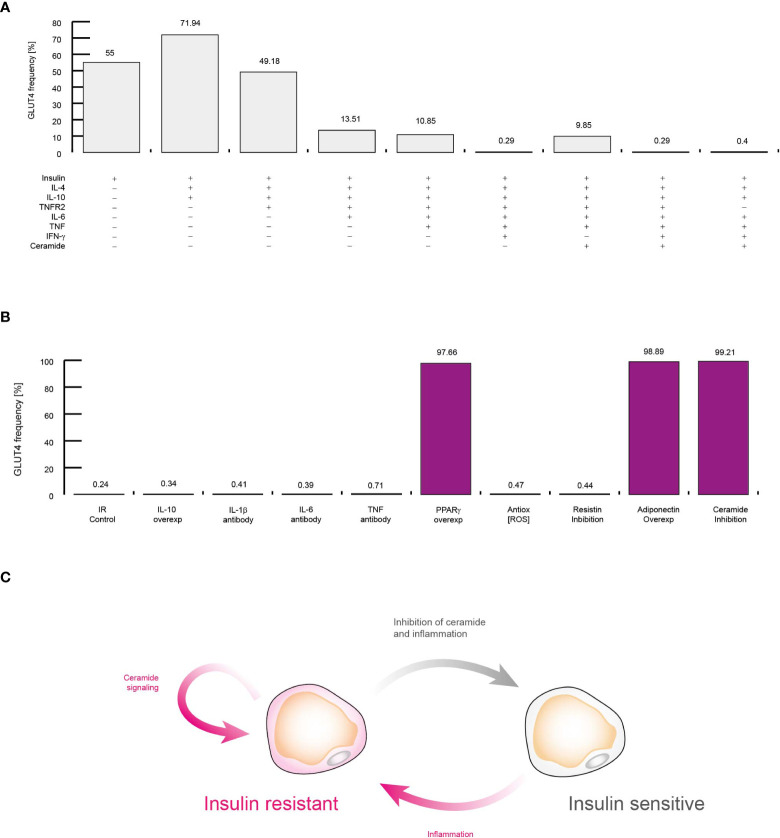
Molecular mechanism that sustains insulin resistance in adipocytes. **(A)** Simulations of controlling the insulin response in different conditions. Each simulation was performed by activating inputs of the adipocyte model (+) in presence of insulin (See methods). **(B)** Simulations of treatments against insulin resistance. Each simulation was performed considering high levels of TNF, IL-6, and IFN-γ. The effect of neutralizing antibodies was simulated by turning off the corresponding node of each target molecule. Over-activation was simulated by turning on the target molecule for all time steps. **(C)** Conceptual model to explain insulin resistance. All data presented in this figure suggest that inflammation alone produce insulin resistance, and intracellular ceramide signaling sustains this pathological condition.

The dataset used to validate our phenotype classification algorithm was obtained by Kanno et al. (27) and is available in Gene Expression Omnibus with the accession number GSE210222. The outcomes of comparing our algorithm to xCell software, is freely available in Data File 1. All the calculations made by the stochastic models to determine the gene expression frequencies, along with the numerical data of **Figures 3**–**8**, are found in Data File 2.

## Results

### The models reproduce the behavior of adipocytes, macrophages and CD4+ T cells

Each model was constructed using data from experimental literature summarized on a gene regulatory network (GRN) (**Supplementary Information**). After that, we applied Boolean formalisms to model each GRN (**Supplementary Information**) to obtain a computational model for all cell types. We analyzed each Boolean model to find stable gene expression patters (i.e., fixed points) that were classified to all phenotypes selected in **Figure 1**. We validated our algorithm to classify attractors to cell phenotypes by comparing its results with xCell software outcomes. After determining that the algorithm works, and correctly identifies the cell phenotypes (Data File 1), we used these attractors to construct computational models based on discrete Markov chains (see Methods and **Supplementary Information**). This type of stochastic models enable predictions of phenotypic distributions (29), that can be validated with flow cytometry available data, or generate novel predictions to be tested in future experiments. Once the models were finished, we tested whether all of them were robust and capable to reproduce the biological aspects of the cell type they represent. To this end, we investigated a series of chemical signals that trigger specific responses on the cell types studied, for example we sought to know the effect of certain combinations of polarizing cytokines, such as IL-2 and IL-4, on the differentiation of CD4+ T lymphocytes. We then used those well characterized conditions to perform stochastic simulations with different levels of randomness (3, 8 and 13% of noise), and we compared the outcomes of models to data of cellular quantifications performed with flow cytometry.

As a result of this procedure, our model of CD4+ T lymphocytes showed that in the absence of stimuli, the dominant phenotype is Th0, as it is observed experimentally (31) (**Figure 3A**). The model also showed that during stimulation of IL-12 and IFN-γ, the dominant phenotype is Th1, while Th2 remains at basal levels, as confirmed by flow cytometry data (31) (**Figure 3B**). The opposite occurs when CD4+ T cells are treated with IL-2 and IL-4, since Th2 phenotype increases while phenotype Th1 is reduced (31) (**Figure 3C**). On the other hand, the model showed that the presence of TGF-β and IL-4 increases the frequency of Th9 phenotype (24) (**Figure 3D**), while combinations of TGF-β and IL-6 increases Th17 phenotype (31) (**Figure 3E**). Interestingly, the model of CD4+ T cells also showed that TGF-β and IL-2 produce Th1R, Th2R and iTreg phenotypes, as it has been observed in previous experimental results (31) (**Figure 3F**). In agreement with experimental data, the model of macrophages showed that the absence of stimuli favors the M0 phenotype (32) (**Figure 3G**), while TLR4 stimulation together with the presence of IFN-γ promote the M1 phenotype (32) (**Figure 3H**). In the same way, the model of macrophages shows that IL-4 and IL-13 polarizes these cells towards M2 phenotype (32) (**Figure 3I**). Similarly, the model of macrophages shows that a combination of GM-CSF, IL-4 and IL-10 polarizes these cells towards M2-TAM phenotype (33) (**Figure 3J**). Regarding the model of adipocytes, it showed that inflammation inducers like CoCl_2_ increases the expression of TNF in these cells, as it has been characterized *in vitro* (34) (**Figure 3K**). Finally, the model of adipocytes showed that TNF reduces the translocation of GLUT4 in presence of insulin, which is responsible of glucose uptake (34) (**Figure 3L**).

The results of the three models and their validation with previous experimental data suggest that they are useful qualitative tools (**Figure 3**). Consequently, we decided to test whether the results obtained with the models could result from random fluctuations or whether the results were statistically significant. To assess the qualitative performance of the models, we compared the phenotypic relationships observed *in vitro* against observations obtained *in silico*. For instance, in the absence of stimuli, Th0 is the dominant phenotype for CD4+ T lymphocytes *in vitro* (31), then we compare whether this phenotype is also dominant in computational simulations made under the same conditions. In positive case, we count this trial as a success, otherwise this trail must be considered as a failure. Based on this rationale, we count the successes and failures obtained for all the microenvironments evaluated and for all the noise levels used in each model. This procedure was done to calculate a probability of success associated with each model, and subsequently a binomial test was performed to determine whether the probability of success of each model is greater than expected by chance or not (**Table 1**). As a result of this procedure, we found the probabilities of success for CD4+ T lymphocytes (p = 2.462e-05), macrophages (p = 0.0002441) and adipocytes (p = 0.0002441) models were significantly higher than chance (**Table 1**). Finally, we tested the quantitative accuracy of each stochastic model by performing a multivariate correlation analysis (**Figure 4**). In all cases, we found that simulated outcomes are strongly correlated with experimental observations, which indicates that these models effectively represent each cell type. Collectively, these results show that all models are robust and accurate to reproduce the biology of adipocytes, macrophages and CD4+ T cells as characterized by different combinations of cytokines and ligands under physiological context.

**Table 1:** Qualitative validation of stochastic models of the VAT cells.

CD4+ T cells				
Inputs (Microenvironments)	Experimental observations	Noise level score*		
		3%	8%	13%
None	Th0 > others	1	1	1
None	Others = 0%	0	0	0
IL-12 + IFN-γ	Th1 > Th2	1	1	1
IL-2 + IL-4	Th2 > Th1	1	1	1
IL-4 + TGF-β	Th9 > Th0	1	1	1
IL-6 + TGF-β	Th17 > Th2	1	1	1
IL-2 + TGF-β	Treg > Th1R	1	1	1
IL-2 + TGF-β	Treg > Th2R	1	1	1
IL-2 + TGF-β	Th1R > Th2R	1	1	1
Trials n = 27, Binomial probability of success = 0.8889, CI: 0.7372 – 1, α = 5%, **p-value = 2.462e-05**				
Macrophages				
Inputs (Microenvironments)	Experimental observations	Noise level score*		
		3%	8%	13%
None	M0 > M2	1	1	1
TLR4 + IFN-γ	M1 > M2	1	1	1
IL-4 + IL-13	M2 > M1	1	1	1
IL-4 + IL-10 + GM-CSF	M2-TAM > M1	1	1	1
Trials n = 12, Binomial probability of success = 1, CI: 0.7791 – 1, α = 5%, **p-value = 0.0002441**				
Adipocytes				
Inputs (Microenvironments)	Experimental observations	Noise level score*		
		3%	8%	13%
None	TNF- > TNF+	1	1	1
Inducer (CoCl_2_)	TNF+ > TNF-	1	1	1
Insulin	GLUT4+ > GLUT4-	1	1	1
Insulin + external TNF	GLUT4- > GLUT4+	1	1	1
Trials n = 12, Binomial probability of success = 1, CI: 0.7791 – 1, α = 5%, **p-value = 0.0002441**				

*1 is assigned for asserts and 0 is assigned for failures. Bold values indicate p-value associated with each one-tailed binomial test.

### Insulin promotes local inflammation in healthy VAT

After validating each model, we focused on simulating the necessary conditions to recreate VAT dynamics in healthy, obese and diabetic patients. To this end, we investigated which were the characteristic cytokines, hormones and chemical signals of VAT in the aforementioned physiological states. It has been reported that VAT of lean patients is characterized by low levels of IL-6, TNF and IL-8 (36). In contrast, relatively high levels of IL-4, IL-10, IL-13 and TGF-β have been seen in these persons (36). Interestingly, the presence of IL-12 and IFN-γ in basal conditions has also been observed (9). In turn, it has been possible to observe *ex vivo* that the adipocytes of lean patients have low levels of expression of the TNF receptor type 2 (TNFR2) (34), together with the absence of ceramides in plasma (37). Using this information, we were able to propose a suitable set of microenvironments to simulate the most likely environments in which VAT cells can be found (**Figure 5A**). Using the aforementioned conditions as reference, our computational models showed that VAT adipocytes from lean subjects in the presence of insulin strongly increase GLUT4 translocation, and some of them can increase TNF and CTGF gene expression (**Figure 5B**).

Regarding macrophages, it is known that several fatty acids such as palmitic acid can activate TLR4 signaling in these cells (38), so it has been reported that such receptors can be activated in VAT (39). Considering these facts, as well as the cytokine profile mentioned above, the stochastic model of macrophages showed that the M2 phenotype is the most predominant cell type in the absence (**Figure 5C**) as well as in the presence of insulin (**Figure 5D**). On the other hand, a lower prevalence of the pro-inflammatory phenotype M1 is also observed (**Figures 5C, D**). In a biological context, it is impossible to directly measure the prevalence of each of the microenvironments *in situ*, in fact, what can be measured in the laboratory is a mixture of all the microenvironments present in the VAT. For this reason, we decided to average the population frequency obtained from each of the simulated microenvironments with or without insulin, in order to observe the qualitative global effect of this hormone on the effector response of macrophages. As a result of this procedure, we found that insulin slightly favors the appearance of M1 phenotype to the detriment of the M2 phenotype (**Figure 5E**).

On the other hand, the absence of insulin promotes anti-inflammatory linages of CD4+ T cells, particularly the Th3 phenotype. In the same way, CD4+ lymphocytes model predicts the prevalence of Th9 population, followed by the phenotypes Th1 and Th2 (**Figure 5F**). However, the presence of insulin increases Th2 phenotype frequency while reducing Th9 population. It is interesting to note that either in the presence or in the absence of insulin; no substantial changes are seen in Th1 population, while there is a slight increase in Th17 population due to insulin stimulation. It should be noted that insulin affected the distribution of T-regulatory lineages, biasing the population balance towards the iTreg environment to the detriment of Th3 (**Figure 5G**). By averaging the microenvironments, as it was done with the macrophage model, it is observed that at a global level, insulin in lean people increases Th1, Th2 and Th17 subpopulations. Similarly, it can be seen that insulin has a negative effect on Th9 and Treg lineages (**Figure 5H**). Finally, we compare the outcomes of our models with *ex vivo* data from lean patients. Herein is observed that Th1 phenotype is more frequent than Th17 phenotype (35) (**Figure 5I**). Similarly, it has been reported that the M2 phenotype is more frequent than the M1 phenotype in lean healthy patients (9) (**Figure 5J**), facts that were reproduced by the models, which in turn validates the models presented here. Collectively, these data suggest that insulin has the property of inducing local inflammation in VAT during normal homeostatic conditions and such conditions emerge from the complex regulatory networks that underlie gene expression of constitutive cells of the VAT, such as CD4+ T cells, macrophages and adipocytes.

### Insulin enhances inflammation and Th17 response in obese patients

Unlike the outcomes observed in VAT of lean and healthy patients, obese patients present a higher expression of IL-6, IL-8 and TNF (36). Similarly, it has been observed that the expression of IL-2 (40) and IL-10 (41) increases, while the IL-4 levels decrease (36). In addition, it has been reported that the infiltration of macrophages and T lymphocytes to the VAT increases (9), which enhances the local inflammation (**Figure 6A**). Considering these data, we use them to reframe the modeled microenvironments in the VAT. As a result of this procedure, the model of adipocytes showed that local inflammation in the VAT induces adipocytes to secrete more CTGF, condition that only occurs when these cells increase their size. In this sense, adipocytes also increased their expression of TNF, contributing to the microenvironment of inflammation (**Figure 6B**). On the other hand, when adipocytes were stimulated with insulin, a reduction in the number of GLUT4-positive cells was observed, and in the same way, the expression of TNF was markedly increased (**Figure 6B**). In absence of insulin (**Figure 6C**), the macrophages presented a pattern in which the M2 phenotype was predominate over the M1 phenotype; but the addition of insulin slightly changed this scenario, causing the M1 phenotype to be slightly increased (**Figures 6D–F**).

Regarding CD4+ T lymphocytes, our stochastic model showed that patients in the absence of insulin, there is a considerable population of pro-inflammatory Th17 lineage. However, insulin drastically increases Th17 phenotype but not Th1 linage (**Figure 6G**). In accordance with our model, the average response observed in CD4+ T lymphocytes showed that insulin particularly favors Th17 phenotype while the Treg phenotype is disfavored (**Figure 6H**). It should be noted that in *ex vivo* models it has been observed that the reduction of Treg cells in obese patients aggravates obesity and leads to insulin resistance (42). Interestingly, our CD4+ T cells model predicts that in response to high insulin levels, regulatory T cells are polarized toward IL-10-producing lineages like Tr1 to the detriment of TGFβ-producing lineages such as Th3 (**Figure 6G**), which is consistent with experimental observations, in which it can be verified that one of the physiological adaptations of obesity is the production of IL-10 (43) and the decrease in TGFβ is associated with the appearance of insulin resistance in obese persons (42). Lastly, we compared the mean frequency of Th1, Th17, M1, and M2 phenotypes of lean, healthy patients versus obese patients (**Figures 6I, J**). This comparison allows us to see that typical inflammation on obesity tends to bias the immune response towards Th17 immunity (35) (**Figure 6I**). On the other hand, the pro-inflammatory M1 phenotype is markedly increased in obese patients (9) (**Figure 6J**). Collectively, these results suggest that insulin promotes the inflammatory process in obese patients towards a Th17 response.

### Ceramides inhibit Th1 response in diabetic patients

The abnormally high level of ceramides in the bloodstream is one of the most distinctive markers of type 2 diabetes (37, 44). Thus, to simulate VAT conditions in obese and diabetic patients, the microenvironments used to simulate the context of obesity were given added high levels of plasma ceramides (**Figure 7A**). Under these conditions, the adipocyte model showed that insulin resistance was maximized in this type of cells (**Figure 7B**). While, in presence or in absence of insulin, the macrophage model showed that M1 phenotype increases 50% of its frequency compared to healthy patients in same conditions (**Figures 7C-E**). On the other hand, the model of CD4+ T cells showed that the immunity was biased towards the Th17 phenotype to the detriment of the other effector responses either with or without insulin (**Figures 7F-H**). Interestingly, our model showed that the presence of ceramides increases the frequencies of FoxP3+ cells such as Th1R, Th2R and iTreg phenotypes, avoiding the inhibition produced by high levels of insulin as it was observed in obese patients (**Figure 7H**). Perhaps this phenomenon is a chronic adaptation to the metabolic imbalance present in diabetics, as has been observed in obese patients (41). This hypothesis might explain why in patients of type 1 diabetes FoxP3+ Treg cells increase at onset of the disease (45). Finally, we compare the data from our simulations against the data obtained *ex vivo* from obese and diabetic patients. The model of CD4+ T cells shows a decrease in Th1 lineage while Th17 population increases, as occurs in real observations (35) (**Figure 7I**), and similarly, the model of macrophages predicts a considerable increase of M1 macrophages in VAT of obese and diabetic patients compared to healthy patients, as it was observed in *ex vivo* data (9) (**Figure 7J**). Together, these results suggest that extracellular ceramides potentiate the inflammatory effect of insulin by stimulating Th17 response to the detriment of other immune responses such as Th1.

### Inflammation triggers intracellular ceramide signaling to induce insulin resistance

Up to this point, our data suggest that the presence of extracellular ceramide is sufficient to alter the normal polarization of cells in the immune system. However, it remains to be determined whether extracellular ceramide is capable of inducing insulin resistance in VAT adipocytes. To investigate this point, we explored several possible scenarios in which some important regulators of adipocytes are blocked or overexpressed in presence of insulin (**Figure 8A**). In agreement with previous reports, our model shows that IL-10 and IL-4 improve insulin sensitivity (46) (**Figure 8A**). Similarly, our simulations showed that the presence of TNFR2 decreases the potential of adipocytes to respond to insulin (34, 47) (**Figure 8A**). Also, our data were also capable to reproduce other biological relevant observations such as high levels of IL-6 (48), TNF (49) and IFN-γ (50) can induce insulin resistance in adipocytes (**Figure 8A**). It should be noted that even without the presence of TNFR2, the context of high levels of inflammation was sufficient to induce insulin resistance in adipocytes (**Figure 8A**). In any case, the extracellular levels of ceramides were not a decisive factor for the appearance of insulin resistance, unlike inflammation (**Figure 8A**).

Next, we carry out a series of simulations to determine the molecular mechanism that sustains insulin resistance and visualize therapeutic strategies to neutralize it. For this purpose, we also carried out a series of simulations in which we maintained constitutively active or inhibited some molecular components that participate in the control of adipocyte gene expression regulation, such as interleukins (**Figure 8B**). Our data showed that in cases of severe inflammation, insulin resistance cannot be restored by IL-10 alone, as it was recently reported (51). In this sense, our simulations showed that therapies based on neutralizing antibodies directed against pro-inflammatory cytokines such as TNF (52) and IL-6 (53) do not improve insulin resistance (**Figure 8B**). In this sense, our simulations showed that antioxidants (54) as well as IL-1β neutralizing antibodies do not increase glucose uptake in response to insulin (55). However, our data pointed out that PPARγ over-activation may improve insulin sensitivity, as it has been observed *in vitro* (56) (**Figure 8B**). In this regard, our data showed that maintaining high levels of adiponectin was enough to restore insulin sensitivity, as it has been report previously (57). This made us think that insulin resistance is supported by intracellular signaling of ceramides, since adiponectin receptors degrade intracellular ceramides when activated (58), then in our last simulation the production of ceramides was suppressed inside adipocytes, thereby restoring insulin sensitivity even in a highly inflammatory setting (59) (**Figure 8B**). Thus, our data showed that inflammation produces insulin resistance through the activation of intracellular ceramide signaling. For their part, once ceramides are activated, they can sustain insulin resistance in adipocytes thanks to their numerous positive feedback loops at the signaling level (**Figure 8C**).

## Discussion

In this paper, we have developed stochastic dynamic network models to explore the complex molecular mechanisms that underlie insulin resistance and the interactions of the immune system with this pathological condition. We modeled regulatory circuits previously characterized for cells of the immune system residing in VAT and adipocytes under contrasting physiological contexts. In this direction, our models showed that insulin has a pro-inflammatory effect not only on cells of the immune system, as previously characterized, but also on adipocytes (**Figure 5B**). In fact, our results suggest that the pro-inflammatory effect on adipocytes works as a homeostatic mechanism to downregulate GLUT4 activity, preventing all adipocytes from absorbing glucose in large quantities (**Figures 5B** and **6B**). In addition, our simulations suggest that adipocytes together with M2 macrophages and Treg cells counteract the temporary induction of inflammation by secreting adiponectin (**Figure 5B**) as well as anti-inflammatory (**Figures 5E, H**) cytokines to maintain metabolic balance in VAT. The homeostatic inflammation produced by adipocytes may explain why there are resident populations of M1 macrophages and Th1 lymphocytes in VAT (9). Moreover, our results show that once a certain limit of inflammation tolerated by VAT is exceeded, adipocytes begin to increase its size. Along with this, adipocytes also increase the secretion of pro-inflammatory cytokines and decrease their levels of adiponectin, leading to obesity (**Figure 6B**). It is important to mention that in this scenario, high insulin levels promote the formation of pro-inflammatory lineages of macrophages and CD4+ T cells, with Th17 and Th1 phenotypes being especially favored to the detriment of Treg cells. On the other hand, the adipocyte model showed that local inflammation decreases insulin sensitivity, which is a pre-diabetic signature. Finally, our models showed that once inflammation increases and lipotoxicity symptoms appear, denoted by high levels of plasmatic ceramide, insulin resistance becomes more intense in adipocytes and the polarization of CD4+ T cells favors Th17 response instead of Th1 immunity (**Figure 7F**). In this scenario, our data suggest that hyperinsulinemia can compensate for the metabolic imbalances produced by inflammation and lipotoxicity, increasing the number of FoxP3+ Treg cells (**Figure 7G**). This compensatory mechanism may explain why an increase in FoxP3+ Treg cells has been observed in patients with type 1 diabetes at the onset of the disease (45).

Regarding the central question of how insulin resistance is originated, our results showed that when the inflammation pathways in adipocytes are activated, ceramide signaling enhances a series of feedback loops that inhibit the pathway of insulin receptor (**Figure 8B**). More importantly, our data showed that it is not possible to eliminate insulin resistance with an anti-inflammatory approach alone (**Figure 8C**). In fact, our computational projections suggested that to alleviate insulin resistance, a combined approach of anti-inflammatory therapy together with inhibitors of intracellular ceramide signaling is needed, because anti-inflammatory actions would control the inflammation of adipocytes as well as the immune system, particularly Th17, Th1 and M1 populations, while ceramide inhibitors would favor the sensitization of insulin-resistant adipocytes. On the other hand, we noticed that the pathways involved in the generation of insulin resistance are present in other cell types that are responsive to this hormone. This suggests that such mechanism could be present in other insulin-responsive cells and insulin resistance could have a broader physiological and evolutionary role. Interestingly, in acute viral infections, high levels of IFN-γ can directly induce insulin resistance in muscle cells, which serves to redirect energy resources towards the immune system, enhancing its effector function (50). It was also been found that in obese mice, this mechanism rapidly leads to diabetes (50). Interestingly, a previous work showed that muscle cells were directly responsible for insulin resistance caused by viral infections instead of VAT (50), which reinforces the hypothesis of homeostatic role of insulin resistance.

**Figure 9:**
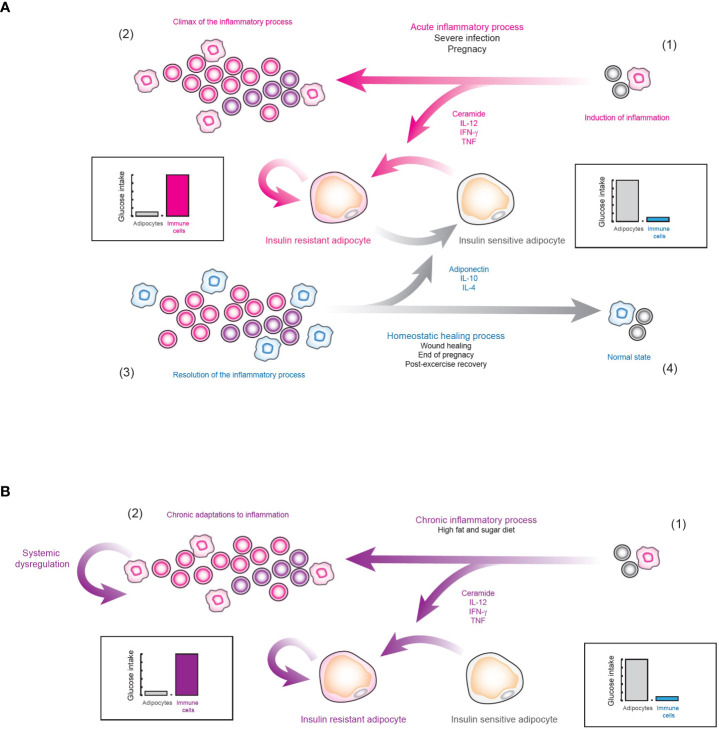
Hypothesis of the physiological role of insulin resistance. **(A)** Insulin resistance as nutrient redirecting mechanism. 1) When a strongly inflammatory stimulus is detected, the systemic release of pro-inflammatory cytokines is favored, which induces insulin resistance in cells responsive to this hormone, such as adipocytes. 2) In this way, the effector function of the immune system is enhanced, in order to neutralize the source of immunogenic signals. 3) After the pro-inflammatory stimulus is controlled, the body inhibits inflammation. 4) As a result, many anti-inflammatory cytokines such as IL-4 and IL-10 are released along with hormones such as adiponectin, which together inhibit inflammation and block intracellular ceramide signaling. Consequently, insulin resistance is eliminated and systemic homeostasis is restored. **(B)** Type 2 diabetes mellitus. 1) As a result of an alteration in the normal inflammatory levels of VAT due to diets rich in sugar and fat, the nutrient redirection mechanism controlled by insulin resistance is locally activated. 2) In consequence, the organism does not process this as a highly inflammatory stimulus. Instead, it processes this type of inflammation as normal VAT behavior, favoring physiological adaptations to chronic inflammation. Consequently, tissue regeneration processes are not activated and this pathological state continues indefinitely, producing type 2 diabetes mellitus.

However, if insulin resistance truly has a physiological role, then why it cannot be reversed in diabetic patients? Perhaps the answer to this question lies in another exceptional physiological condition, pregnancy. In general, during the first and third trimesters of pregnancy, the secretion of pro-inflammatory cytokines such as TNF is strongly increased (60, 61). TNF is capable of inducing insulin resistance in adipocytes (62), vascular smooth muscle cells (63), and skeletal muscle cells (64). In fact, some overweight and obese women, can develop gestational diabetes (65), just as it was observed with viral infections. However, after childbirth, the secretion of adiponectin (66) and anti-inflammatory cytokines such as IL-10 is increased (67), which eventually allows the mother to overcome her gestational diabetes condition. Thus, we hypothesize that strong and exceptional inflammatory conditions such as pregnancy or acute viral infections may induce generalized insulin resistance (50, 68–71), in order to strengthen the effector function of the immune system, and if the patient is pre-diabetic or obese, then they could develop a temporary diabetes (**Figure 9A**). But, after the inflammatory process is over, the organism will activate wound healing processes which will increase the number of anti-inflammatory cytokines as well as adiponectin, removing the insulin resistance and temporary healing insulin resistance and diabetes (**Figure 9A**). Nevertheless, in the case of type 2 diabetes mellitus, caused entirely by inflammation in VAT (50), the nutrient redirection mechanism would be activated without having an exceptional inflammatory context, as occurs with pregnancy and acute viral infections (**Figure 9B**). In fact, our data suggest that in the latter case the organism would not even detect a highly inflammatory stimulus, as occurs with pregnancy and viral infections, since VAT needs local inflammation to function normally (**Figure 9B**). Consequently, anti-inflammatory mechanisms are not be activated and the organism gradually adapts to the local inflammation until type 2 diabetes mellitus appears (**Figure 9B**). If our hypothesis is true, maybe insulin resistance and diabetes could be cured by inducing the release of anti-inflammatory cytokines and adiponectin with exercise (72), along with anti-inflammatory therapy, ceramide-inhibiting drugs, and a rigorous diet. Indeed, exercise has been reported to practically eliminate the risk of developing gestational diabetes (73), and to improve the condition of diabetic patients (74). In any case, more research is required to alleviate insulin resistance and type 2 diabetes mellitus and further understand the complex molecular mechanisms underlying insulin resistance and health-related conditions.

## Data availability statement

The datasets presented in this study can be found in online repositories. The names of the repository/repositories and accession number(s) can be found in the article/**Supplementary Material**.

## Author contributions

AB performed computational modeling, and software implementation. JT-M collected experimental data from literature. EA-B supervised, and provided resources. All authors contributed to the article and approved the submitted version.
