# Supplementary material for: Molecular tracking of insulin resistance and inflammation development on visceral adipose tissue
Source: Front Immunol. 2023 Mar 21;14:1014778. doi: 10.3389/fimmu.2023.1014778 (PMC10070947; doi:10.3389/fimmu.2023.1014778)
Supplement: Supplementary file 3 [file DataSheet_3.docx]

Supplementary Material

**Molecular Tracking of Insulin Resistance and Inflammation Development on Visceral Adipose Tissue**

**Antonio Bensussen^*^, José Antonio Torres-Magallanes, Elena Roces de Álvarez-Buylla***

Laboratorio de Neuroendocrinología, Centro Universitario de Investigaciones Biomédicas, Universidad de Colima, Colima 28040, México

*** Correspondence:**AB: [antonio.bensussen@gmail.com](mailto:antonio.bensussen@gmail.com)

ERAB: [rab@ucol.mx](mailto:rab@ucol.mx)

CONTENT:

[Gene regulatory networks 2](#_Toc107141669)

[Algorithm to reduce networks 5](#_Toc107141670)

[Biological evidences that support reduced networks 8](#_Toc107141671)

[Calculating attractors for each network 15](#_Toc107141672)

[Details of stochastic modeling for each GRN 20](#_Toc107141673)

[Supplementary references 22](#_Toc107141674)

# Gene regulatory networks

In order to construct all network representation of macrophages and adipocytes, we reviewed literature sources to extract particularities about signaling and differentiation processes of those cells(1–6). After that, we used the Human Protein Atlas (proteinatlas.org)(7) to verify the expression levels of signature genes of each cell type. We then complemented the information collected, by consulting all interactions reported in Kyoto Encyclopedia of Genes and Genomes, KEGG(8–10). We present the summary of the information collected for adipocytes and macrophages in Supplementary Figures 1 and 2. On the other hand, to construct the network of CD4+ T lymphocytes, we used the network published by Martínez-Sánchez *et al* (11) as a base of this work, and we added new reported interactions related to the Th9 phenotype(12).


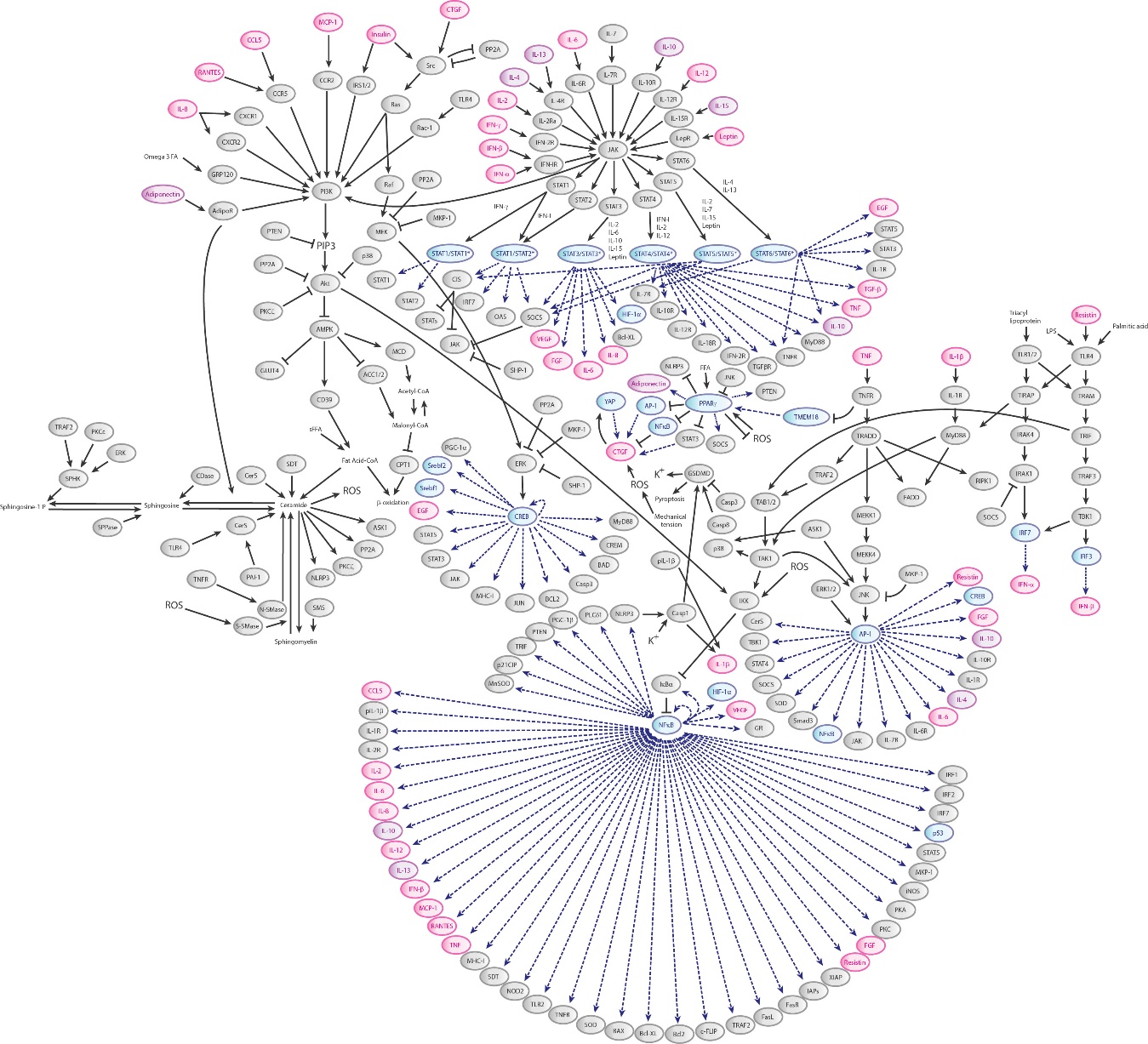


**Supplementary Figure 1**. In this figure, we present the entire GRN of adipocytes presented in the VAT. Pink nodes represent pro-inflammatory molecules, such as IL-6 and resistin. Purple nodes represent anti-inflammatory molecules such IL-10 and Adiponectin. Blue nodes represent master transcriptional factors. Grey nodes represent other molecules presented in adipocytes. Solid black arrows represent direct interactions and dotted blue arrows represent transcriptional activation.


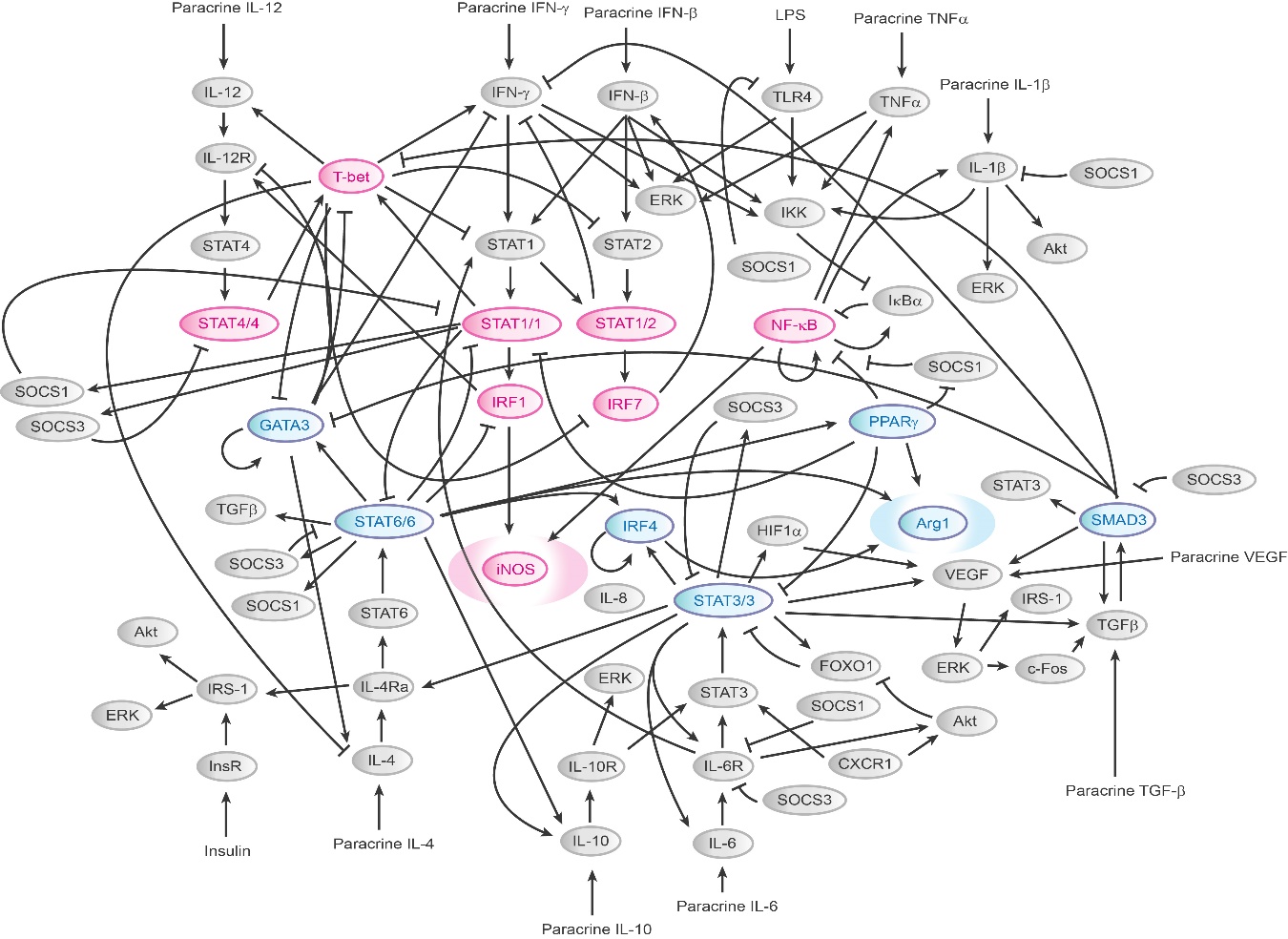


**Supplementary Figure 2**. In this figure, we present the entire GRN of resident macrophages in the VAT. Pink nodes represent pro-inflammatory molecules involved in the M1 program, like T-bet and IRF1. Blue nodes represent anti-inflammatory molecules involved in the M2 program, such as the homodimer of STAT6. Pink shadow is used to highlight iNOS, the main marker of the M1 program while blue shadow is used to highlight Arg1, the main marker of the M2 program. Grey nodes represent other molecules presented in macrophages. Solid black arrows represent direct interactions between the nodes of the network.

# Algorithm to reduce networks

After we obtain experimental information about adipocytes and resident macrophages of the VAT, we used Boolean formalisms to translate this information in logic expressions. In general terms, such expressions assumes that all genes, proteins, RNAs or metabolites of each network can be expressed depending on its presence or absence. This binary approach allows to simplify biological complexity by setting 1 for “activation” and 0 for “inactivation” of every component of the network. Moreover, the current state of all nodes of the network, depends on the activation or inhibition of its regulators. In other words:

$$y_{i}\left( t+\Delta t \right)=g_{i}\left( y_{1}\left( t \right),y_{2}\left( t \right),\ldots,y_{k}\left( t \right) \right)$$

Where *k* represents the number of regulators of the $y_{i}$ node, and $g_{i}$ is the logic function that determine the current state of $y_{i}$ based on the state of its regulators $y_{1}, y_{2}, \ldots, y_{k}$. For example, consider activation of STAT3 by IL-6. In this example, external IL-6 must interact with receptor to activate JAK1/2, which in turn will activate STAT3. Considering these processes, we might represent the activation of IL-6 receptor as follows:

$$IL6R\left( t+1 \right)=IL6^{*}$$

Then, activation of JAK1/2 by IL-6R can be represented as:

$$JAK\left( t+1 \right)=IL6R(t)$$

And finally, we can represent activation of STAT3 by JAK1/2 as follows:

$$STAT3\left( t+1 \right)=JAK\left( t \right)$$

We applied the same reasoning for all interactions of our networks. Finally, to simplify the complexity generated by all interactions reported for each network, we used a reduction method developed and tested by Villarreal and coworkers (13). This method searches for linear interactions between nodes that converge to highly connected nodes with non-linearities, such as feedforward motives, including negative and positive feedback loops. For instance, let the nodes $\left\{ y_{1}, y_{2}, y_{3}, \ldots,y_{n-1}, y_{n} \right\}$ be linearly related from $y_{1}$ to $y_{n}$, where $y_{n}$ is a highly connected node i.e.

$$y_{1}\to y_{2}\to y_{3}\to\ldots\to y_{n-1}\to y_{n}$$

Applying transitivity for this set of nodes, we might reduce this linear sequence as follows:

$$y_{1}\to y_{n}$$

This reduction can only be applied if all interactions between $y_{2}$ and $y_{n-1}$ are linear. To illustrate how we applied this method to reduce the network of adipocytes and macrophages, let’s consider the activation pathway the activation pathway of T-bet in macrophages mediated by IL-12. Initially, extracellular IL-12 interacts with its receptor, to activate JAK2, which in turn activates STAT4 by phosphorylation. Once STAT4 is phosphorylated, this protein creates a homodimer that is able to activate T-bet transcription in the nucleus. Translating all these interactions as logic rules, we might obtain:

$$IL12R\left( t+1 \right)=IL{12}^{*}$$

$$JAK2\left( t+1 \right)=IL12R\left( t \right)$$

$$STAT4\left( t+1 \right)=JAK2\left( t \right)$$

$$STAT4.STAT4\left( t+1 \right)=STAT4\left( t \right)$$

$$TBET\left( t+1 \right)=STAT4.STAT4\left( t \right)$$

Applying transitivity of these interactions, we can obtain:

$$TBET\left( t+1 \right)=IL{12}^{*}$$

We applied the same procedure to all interactions to the networks of adipocytes and macrophages. Regarding CD4+ T cells network, Martínez-Sánchez et al(11) previously had applied the same method of reduction, and we only added nodes related to the Th9 phenotype. In the following tables, we present all reduced networks that we used in this work.

**Supplementary Table 1: Reduced network of adipocytes**

| **Node** | **Logic rule** |  |
| --- | --- | --- |
| $IL1\beta$ | $\left( Akt\vee TNF\vee Resistin \right)\wedge\neg PPAR\gamma$ |  |
| $IL6$ | $\left( Akt\vee TNF\vee Resistin\vee IL6^{*} \right)\wedge\neg PPAR\gamma$ |  |
| $IL8$ | $\left( Akt\vee TNF\vee Resistin \right)\wedge\neg PPAR\gamma$ |  |
| $TNF$ | $\left( Akt\vee\left( TNF\wedge TNFR2 \right)\vee\left( TNF^{*}\wedge TNFR2 \right)\vee Resistin \right)\wedge\neg PPAR\gamma$ |  |
| $IL10$ | $Akt\vee IL4\vee IL10\vee IL{10}^{*}$ |  |
| $IL4$ | $IL4\vee IL10\vee IL4^{*}$ |  |
| $AP1$ | $Insulin\wedge\neg PPAR\gamma$ |  |
| $PPAR\gamma$ | $\left( IL10\vee IL4 \right)\wedge\neg\left( TNF\vee IL6\vee IFN\gamma\right)$ |  |
| $CTGF$ | $ROS\vee\left( CTGF\wedge CTGFR \right)$ |  |
| $Akt$ | $Insulin\wedge\neg Ceramide$ |  |
| $Resistin$ | $\left( AP1\wedge TNF \right)\wedge\neg PPAR\gamma$ |  |
| $Adiponectin$ | $PPAR\gamma\wedge\neg TNF$ |  |
| $Ceramide$ | $\left( sFFA\vee ROS\vee TNF\vee Ceramide^{*} \right)\wedge\neg Adiponectin$ |  |
| $ROS$ | $Ceramide\wedge\left( Pressure\vee\neg PPAR\gamma\right)$ |  |
| $sFFA$ | $Ceramide\wedge\neg Akt$ |  |
| $GLUT4$ | $Akt\wedge\neg Ceramide$ |  |

*Here, we used “$\wedge$, $\vee, \neg$” to represent the Boolean operators “AND”, “OR” and “NOT” respectively. The network inputs are: $IL4^{*}$, $IL6^{*}$, $IL{10}^{*}$, $TNF^{*}$, $Ceramide^{*}$, $Pressure$, $TNFR2$, $CTGFR$, $IFN\gamma$, and $Insulin$

**Supplementary Table 2: Reduced network of macrophages**

| **Node** | **Logic rule** |  |
| --- | --- | --- |
| $IFN\gamma$ | $IFN\gamma^{*}\wedge\neg TGF\beta$ |  |
| $IFN\beta$ | $\left( IFN\beta^{*}\vee IFN\beta\right)\wedge\neg\left( TGF\beta\vee IFN\gamma\right)$ |  |
| $Tbet$ | $\left( \left( IL12\vee IL{12}^{*} \right)\wedge\left( IFN\beta\vee IFN\gamma\vee IL6 \right) \right)\wedge\neg\left( TGF\beta\vee GATA3 \right)$ |  |
| $iNOS$ | $\left( IFN\gamma\vee IFN\beta\right)\wedge\left( IL1\beta\vee TNF\vee IL6\vee TLR4 \right)\vee GMCSF$ |  |
| $TNF$ | $\left( TNF^{*}\vee TNF\vee TLR4\vee IL1\beta\vee IL6\vee Insulin \vee Ceramide^{*} \right)\wedge\neg IL10\wedge\neg IL4$ |  |
| $TLR4$ | $\left( Ceramide^{*}\vee LPS \right)\wedge\neg IL4$ |  |
| $IL1\beta$ | $\left( IL1\beta^{*}\vee IL1\beta\vee TNF\vee TLR4\vee IL6 \right)\wedge\neg IL10\wedge\neg IL4$ |  |
| $IL6$ | $IL6^{*}\vee IL6\vee IL1\beta\vee TNF\vee TLR4\vee TGF\beta$ |  |
| $IL10$ | $\left( IL{10}^{*}\vee IL10\vee TLR4\vee IL6\vee TGF\beta\right)\wedge\neg\left( IFN\beta\vee IFN\gamma\right)$ |  |
| $IL4$ | $\left( IL4^{*}\vee GATA3 \right)\wedge\neg Tbet$ |  |
| $IRF4$ | $\left( IL4\vee IL6\vee IRF4\vee GMCSF \right)\wedge\neg\left( TLR4\vee TNF\vee IL1\beta\right)$ |  |
| $Arg1$ | $IRF4\wedge IL4$ |  |
| $GATA3$ | $\left( IL4\vee GATA3 \right)\wedge\neg\left( Tbet\vee TGF\beta\right)$ |  |
| $TGF\beta$ | $\left( TGF\beta^{*}\vee TGF\beta\vee IL4\vee IL6\vee IL10 \right)\wedge\neg\left( IFN\beta\vee IFN\gamma\vee TLR4\vee TNF \right)$ |  |

*Here, we used “$\wedge$, $\vee, \neg$” to represent the Boolean operators “AND”, “OR” and “NOT” respectively. The network inputs are: $IFN\gamma^{*}$, $IFN\beta^{*}$, $TGF\beta^{*}$, $TNF^{*}$, $IL1\beta^{*}$, $IL4^{*}$, $IL6^{*}$, $IL{10}^{*}$, $IL{12}^{*}$, $Ceramide^{*}$, $LPS$, $GMCSF$ and $Insulin$

**Supplementary Table 3: Reduced network of CD4+ T cells**

| **Node** | **Logic rule** |  |
| --- | --- | --- |
| $Tbet$ | $\left( IFN\gamma\vee\left( IL12\wedge\neg\left( IL6\vee IL4\vee IL10 \right) \right) \right)\vee\left( Tbet \right)\wedge\neg\left( IL6\vee IL4\vee GATA3 \right)$ |  |
| $IFN\gamma$ | $\left( IFN\gamma^{*}\vee\left( \left( IFN\gamma\vee Tbet \right)\wedge\neg\left( GATA3\vee TGF\beta\right) \right) \right)\wedge\neg\left( IL4\vee IL6\vee IL10\vee Ceramide^{*} \right)$ |  |
| $GATA3$ | $\left( (IL2\wedge IL4)\vee GATA3 \right)\wedge\neg\left( Tbet\vee TGF\beta\vee IFN\gamma\vee IL6\vee PU.1 \right)$ |  |
| $IL2$ | $\left( IL2^{*}\vee\left( IL2\wedge\neg FoxP3 \right) \right)\wedge\neg\left( IFN\gamma\vee IL6\vee\left( IL10\wedge\neg FoxP3 \right) \right)$ |  |
| $IL4$ | $\left( IL4^{*}\vee\left( GATA3\wedge\left( IL2\vee IL4 \right) \right)\wedge\neg Tbet \right)\wedge\neg\left( IFN\gamma\vee IL6\vee PU.1 \right)$ |  |
| $ROR\gamma T$ | $\left( TGF\beta\wedge IL6 \right)\wedge\neg\left( Tbet\vee GATA3\vee FoxP3 \right)$ |  |
| $IL6$ | $\left( ROR\gamma T\vee IL6\vee IL6^{*} \right)\wedge\neg\left( IFN\gamma\vee IL2\vee IL4\vee IL10 \right)$ |  |
| $FoxP3$ | $\left( IL2\wedge\left( Ceramide^{*}\vee TGF\beta\vee FoxP3 \right) \right)\wedge\neg\left( ROR\gamma T\vee IL6\vee PU.1 \right)$ |  |
| $TGF\beta$ | $TGF\beta^{*}\vee\left( TGF\beta\vee FoxP3\wedge\neg IL6 \right)$ |  |
| $IL10$ | $IL{10}^{*}\vee\left( IL10\wedge\left( IFN\gamma\vee TGF\beta\vee IL6\vee IL{27}^{*}\vee GATA3 \right)\wedge\neg\left( Insulin\vee Ceramide^{*} \right) \right)$ |  |
| $PU.1$ | $\left( TGF\beta\vee IL6\vee PU.1 \right)\wedge\neg IL2$ |  |
| $IL9$ | $\left( \left( IL4^{*}\vee IL4 \right)\wedge PU.1 \right)\wedge\neg Tbet$ |  |

*Here, we used “$\wedge$, $\vee, \neg$” to represent the Boolean operators “AND”, “OR” and “NOT” respectively. The network inputs are: $IFN\gamma^{*}$, $TGF\beta^{*}$, $IL2^{*}$, $IL4^{*}$, $IL6^{*}$, $IL{10}^{*}$, $IL{12}^{*}$, $IL{27}^{*}$, $Ceramide^{*}$ and $Insulin$

# Biological evidences that support reduced networks

Logical reduction of Boolean networks is a mathematical procedure that generates short networks that might present interactions between nodes that are not directly reported in literature. For instance, suppose that a reduced network shows that IL-12 inhibits GATA3. In this case, there are no direct interactions between those nodes, however, experimental literature pointed out the repressive effect of IL-12 over GATA3, since such interleukin activates STAT4, which activates T-bet and this protein directly downregulates GATA3 expression. Therefore, using these biological arguments reinforces the validity of network reductions. Here, we present experimental bases that sustains our reduced Boolean networks.

Experimental support of adipocytes network

It has been documented adipocytes express many pro-inflammatory cytokines, such as IL-1b(14), IL-6(15), IL-8(16), and TNF(4). Regarding IL-1b, its activation depends on Akt induction of NF-kB(17,18), as well as TNF(19) and Resistin(20) signaling cascades. On the other hand, it has been reported that IL-1b synthesis is abrogated by PPARg(21). Then, we can represent this information as follows:

$$IL1\beta\left( t+1 \right)=\left( Akt\left( t \right)\vee TNF\left( t \right)\vee Resistin\left( t \right) \right)\wedge\neg PPAR\gamma\left( t \right)$$

In similar way, it has been observed that Akt(22), TNF(23) and Resistin(24) are needed for IL-6 activation through activation of NF-kB(25). Other observations pointed out that external IL-6 is able to increase the expression of IL-6 in adipocytes(26), but this pro-inflammatory signal is downregulated by PPARg activity(27). This can be summarized as follows:

$$IL6\left( t+1 \right)=\left( Akt\left( t \right)\vee TNF\left( t \right)\vee Resistin\left( t \right)\vee IL6^{*} \right)\wedge\neg PPAR\gamma\left( t \right)$$

Regarding IL-8 activation, it has been reported a similar behavior of IL-6, except by its own self activation. This means that Akt(22), TNF(23) and Resistin(24) activates IL-8 expression in adipocytes through NF-kB(28), while PPARg(29) inhibits this cytokine, in other words:

$$IL8\left( t+1 \right)=\left( Akt\left( t \right)\vee TNF\left( t \right)\vee Resistin\left( t \right) \right)\wedge\neg PPAR\gamma\left( t \right)$$

On the other hand, it has been observed that TNF expression is enhanced by increasing TNFR2 on adipocytes’ surface(4). In this regard, adipocytes are highly sensitive to TNF(30). Similarly, TNF can be promoted by Akt signaling(22) as well as by Resistin(24) through NF-kB activation(31), and it is inhibited by PPARg(29), that is:

$$TNF\left( t+1 \right)=\left( Akt\left( t \right)\vee\left( TNF\left( t \right)\wedge TNFR2 \right)\vee\left( TNF^{*}\wedge TNFR2 \right)\vee Resistin\left( t \right) \right)\wedge\neg PPAR\gamma\left( t \right)$$

Adipocytes are also able to secrete anti-inflammatory cytokines, such as IL-4(32) and IL-10(32). Concerning to the former one, IL-10 can be induced either by its own signaling through STAT3 activation(33,34), or by IL-4 signaling(35) as well as by Akt activity(22) through NF-kB(36) activation, which implies that:

$$IL10\left( t+1 \right)=Akt\left( t \right)\vee IL4\left( t \right)\vee IL10\left( t \right)\vee IL{10}^{*}$$

About IL-4 activation, it has been documented that in adipocytes it depends on its own signaling(37) as well as IL-10 through STAT3(33) stimulation, which is involved in STAT6 activation as well as the expression of IL-4R(38). In other words:

$$IL4\left( t+1 \right)=IL4\left( t \right)\vee IL10\left( t \right)\vee IL4^{*}$$

Transcriptional factors play an important role orchestrating the entire functioning of cells, and to adipocytes, AP1 can be stimulated by Insulin signaling(39), and it can be downregulated by PPARg activity(40), i.e.,

$$AP1\left( t+1 \right)=Insulin\wedge\neg PPAR\gamma\left( t \right)$$

In regard of PPARg, its activation can be stimulated by anti-inflammatory cytokines like IL-4(41) and IL-10(42). On the contrary, pro-inflammatory cytokines such as TNF through NF-kB activation(43), IL-6(44) and IFN-g(45), have an opposite effect on PPARg, that is:

$$PPAR\gamma\left( t+1 \right)=\left( IL10\left( t \right)\vee IL4\left( t \right) \right)\wedge\neg\left( TNF\left( t \right)\vee IL6\left( t \right)\vee IFN\gamma\right)$$

CTGF is an important reporter gene of the physiological state of adipocytes, since it has been documented that such protein is highly expressed in hypertrophic adipocytes(46), which mainly occurs under obesity. It is noteworthy to say that reactive oxygen species (ROS) increase CTCG expression(47), as well as its own receptor, CTGFR(47). In other words:

$$CTGF\left( t+1 \right)=ROS\left( t \right)\vee\left( CTGF\left( t \right)\wedge CTGFR \right)$$

There are many signals that induce Akt activation, but considering adipose tissue context, insulin is a remarkable stimulator of Akt functions inside adipocytes(48). On the contrary, it has been observed that intracellular ceramides may inhibit Akt functions in different ways(49). Rewriting this information, we can say that:

$$Akt\left( t+1 \right)=Insulin\wedge\neg Ceramide\left( t \right)$$

Adipocytes are also able to produce their own specific cytokines, better known as adipokines, such as Resistin(20) and Adiponectin(50). There are still controversies about the physiological role of such adipokines, but now is clear that in the majority of cases Resistin has a pro-inflammatory function(20), while Adiponectin has an opposite function(14). Concerning Resistin, it has been found that its expression depends on the presence of AP1 transcriptional activity(51) as well as TNF signaling(52), and it is downregulated by PPARg(53). This means that:

$$Resistin\left( t+1 \right)=\left( AP1\left( t \right)\wedge TNF\left( t \right) \right)\wedge\neg PPAR\gamma\left( t \right)$$

On the contrary, it has been reported that Adiponectin needs PPARg activity(54) to be expressed, and is downregulated by TNF(55). In other words:

$$Adiponectin\left( t+1 \right)=PPAR\gamma\left( t \right)\wedge\neg TNF\left( t \right)$$

Recent works highlighted the importance of intracellular ceramides to ensure the functioning of adipocytes(49,56). In this sense, it has been reported that intracellular ceramides might increase due to high levels of ROS(49,56), the increase of free fat acids(49,56), the presence of TNF(49,56) as well as extracellular ceramides. On the other hand, it has been noted that Adiponectin induces intracellular degradation of ceramides(56), inhibiting its effects on adipocytes signaling. Thus, such information can be abstractly represented as follows:

$$Ceramide\left( t+1 \right)=\left( sFFA\left( t \right)\vee ROS\left( t \right)\vee TNF\left( t \right)\vee Ceramide^{*} \right)\wedge\neg Adiponectin\left( t \right)$$

About ROS, it has been observed that their levels may be increased by the presence of intracellular ceramides(57) and either by mechanical pressure generated in hypertrophic adipocytes(58) or by the absence of PPARg activity(59). This means that:

$$ROS\left( t+1 \right)=Ceramide\left( t \right)\wedge\left( Pressure\vee\neg PPAR\gamma\left( t \right) \right)$$

It has been observed that intracellular ceramides increase the presence of free fat acids inside adipocytes(49), which is healed by Akt activity(60). In other words:

$$sFFA\left( t+1 \right)=Ceramide\left( t \right)\wedge\neg Akt\left( t \right)$$

Finally, GLUT4 is translocated to the adipocytes’ surface when Akt signaling is active(61), and it is not hindered by intracellular ceramides(60). That is:

$$GLUT4\left( t+1 \right)=Akt\left( t \right)\wedge\neg Ceramide\left( t \right)$$

Experimental support of macrophages network

There is a notorious population of macrophages in the visceral adipose tissue. These resident macrophages may be polarized whether to M1 pro-inflammatory phenotype or to M2 anti-inflammatory phenotype. Such phenotypes are characterized by secretion of specific cytokines that contribute to regulate the immune response. One of the most important pro-inflammatory cytokines is IFN-g, which is produced by M1 macrophages(62), and its activation depends on the presence of external pro-inflammatory signals like IFN-g itself(62). It has been observed that expression of IFN-g is reduced due to anti-inflammatory cytokines like TGF-b(63). In other words:

$$IFN\gamma\left( t+1 \right)=IFN\gamma^{*}\wedge\neg TGF\beta\left( t \right)$$

On the other hand, IFN-b is an important cytokine that controls resolution of inflammation by macrophages(64). The expression of this cytokine can be triggered by itself(64) and other cytokines such as IFN-g trough STAT1-dependent activation of IRF7(65–67) and TGF-b(68) produces opposite effects, that is:

$$IFN\beta\left( t+1 \right)=\left( IFN\beta^{*}\vee IFN\beta\left( t \right) \right)\wedge\neg\left( TGF\beta\left( t \right)\vee IFN\gamma\left( t \right) \right)$$

T-bet is a key transcription factor that regulates the expression of several cytokines of the M1 program(69,70). T-bet can be activated by IL-12 signaling(71,72), and another cytokine that directly or indirectly activates STAT1 such as IFN-g(69), IFN-b(73,74) and IL-6(75) through STAT1 pathway(76). On the contrary, GATA3(77) and TGF-b signaling(78) inhibit T-bet activation, which can be summarized as follows:

$$Tbet\left( t+1 \right)=\left( \left( IL12\left( t \right)\vee IL{12}^{*} \right)\wedge\left( IFN\beta\left( t \right)\vee IFN\gamma\left( t \right) \vee IL6\left( t \right) \right) \right)\wedge\neg\left( TGF\beta\left( t \right)\vee GATA3\left( t \right) \right)$$

The enzymatic activity of iNOS is the most characteristic marker of the M1 program(79). Such enzyme might be activated by pro-inflammatory cytokines like GM-CSF(80), IFN-g(81), IFN-b(82), TNF(83), IL-1b(84) and IL-6 through NF-kB activation(85), including by the signaling of TLR4(86). In other words:

$$iNOS\left( t+1 \right)=\left( IFN\gamma\left( t \right)\vee IFN\beta\left( t \right) \right)\wedge\left( IL1\beta\left( t \right)\vee TNF\left( t \right)\vee IL6\left( t \right)\vee TLR4\left( t \right) \right)\vee GMCSF$$

Concerning to TNF, this cytokine can be activated either by external TNF(87), or by others pro-inflammatory cytokines that activates NF-kB like IL-1b(88) and IL-6(44), as well as by the signaling of TLR4(89). On the other hand, anti-inflammatory cytokines like IL-4(90) or IL-10(91) might abrogate the secretion of TNF. In summary:

$$TNF\left( t+1 \right)=\left( TNF^{*}\vee TNF\left( t \right)\vee TLR4\left( t \right)\vee IL1\beta\left( t \right)\vee IL6\left( t \right)\vee Insulin \vee Ceramide^{*} \right)\wedge\neg IL10\left( t \right)\wedge\neg IL4\left( t \right)$$

About TLR4 activation, it mainly depends on the presence of antigens like LPS(92) or by external signals like ceramides(93), but anti-inflammatory stimuli like IL-4(94) can inhibit TLR4 signaling pathway, i.e.,

$$TLR4\left( t+1 \right)=\left( Ceramide^{*}\vee LPS \right)\wedge\neg IL4\left( t \right)$$

As occurs with TNF, the activation of IL-1b is ruled by NF-kB(95), which can be triggered by IL-1b itself(88), as well as by TNF(87), IL-6(44) and other pro-inflammatory pathways like TLR4(92) stimulation. Nevertheless, the expression of IL-1b can be silenced in presence of anti-inflammatory cytokines like IL-4(96) and IL-10(97). In other words:

$$IL1\beta\left( t+1 \right)=\left( IL1\beta^{*}\vee IL1\beta\left( t \right)\vee TNF\left( t \right)\vee TLR4\left( t \right)\vee IL6\left( t \right) \right)\wedge\neg IL10\left( t \right)\wedge\neg IL4\left( t \right)$$

The same is true for IL-6, since its activation depends on NF-kB(25) and, as well as other pro-inflammatory cytokines such as IL-1b(88) and TNF(87). However, IL-6 can be activated by TGF-b(98), that is:

$$IL6\left( t+1 \right)=IL6^{*}\vee IL6\left( t \right)\vee IL1\beta\left( t \right)\vee TNF\left( t \right)\vee TLR4\left( t \right)\vee TGF\beta\left( t \right)$$

Regarding the M2 anti-inflammatory program, it is characterized by the expression of specific cytokines like IL-4, IL-10 and TGF-b(99), as well as by specific transcription factors like GATA3(100) and IRF4(99), including the notable enzyme arginase 1 (Arg1)(79). Concerning IL-10 activation, it can be stimulated by IL-10 itself through STAT3 activation(33,34), as well as by cytokines like TGF-b(101) and IL-6 by STAT3 activation(75). It is noteworthy to say that TLR4 can also activate the expression of IL-10(102), inducing an alternative activation of macrophages (i.e., the M2 program). On the other hand, IL-10 activity can be abrogated by pro-inflammatory cytokines such as IFN-g(103) or IFN-b(104). Thus, we can summarize this information as follows:

$$IL10\left( t+1 \right)=\left( IL{10}^{*}\vee IL10\left( t \right)\vee TLR4\left( t \right)\vee IL6\left( t \right)\vee TGF\beta\left( t \right) \right)\wedge\neg\left( IFN\beta\left( t \right)\vee IFN\gamma\left( t \right) \right)$$

In this sense, IL-4 can be activated either by STAT6(105) (induced by itself(105)) or by GATA3(105) activity. Moreover, IL-4 can be precluded by T-bet transcriptional activity(106), which can be expressed as:

$$IL4\left( t+1 \right)=\left( IL4^{*}\vee GATA3\left( t \right) \right)\wedge\neg Tbet\left( t \right)$$

Concerning IRF4, this is a signature transcription factor of the M2 program(107), which can be activated either by itself(108), or by IL-4(108), GM-CSF(108) and IL-6(109). IRF4 activity is hindered by IRF5(110), which activation depends on pro-inflammatory signals like TLR4 activation, TNF and IL-1b(111). Then, we can summarize this information as follows:

$$IRF4\left( t+1 \right)=\left( IL4\left( t \right)\vee IL6\left( t \right)\vee IRF4\left( t \right)\vee GMCSF \right)\wedge\neg\left( TLR4\left( t \right)\vee TNF\left( t \right)\vee IL1\beta\left( t \right) \right)$$

The enzyme Arg1 is the most distinctive marker of the M2 program(79), and its activation can be produced only when IRF4(111) is activated and there are high levels of IL-4(111) in the environment. In other words:

$$Arg1\left( t+1 \right)=IRF4\left( t \right)\wedge IL4\left( t \right)$$

As occurs with IRF4, GATA3 is an important transcription factor that regulates several genes implicated in the M2 program(100). To activate this transcription factor, it is required either its presence(105) or IL-4 signaling(105), as well as the absence of T-bet(77) and TGF-b(112). Thus:

$$GATA3\left( t+1 \right)=\left( IL4\left( t \right)\vee GATA3\left( t \right) \right)\wedge\neg\left( Tbet\left( t \right)\vee TGF\beta\left( t \right) \right)$$

Finally, the activation of TGF-b depends on AP-1 levels(113), which can be enhanced by the presence of TGF-b itself(114), or by the presence of other signals like IL-4(115), IL-10(116), IL-6(117) and the absence of IRF5(118) which can be activated by strong pro-inflammatory signals like TNF(119), IFN-g¸ IFN-b as well as TLR4 signaling(120). Therefore, we can express this information as follows:

$$TGF\beta\left( t+1 \right)=\left( TGF\beta^{*}\vee TGF\beta\left( t \right)\vee IL4\left( t \right)\vee IL6\left( t \right)\vee IL10\left( t \right) \right)\wedge\neg\left( IFN\beta\left( t \right)\vee IFN\gamma\left( t \right)\vee TLR4\left( t \right)\vee TNF\left( t \right) \right)$$

Experimental support of CD4+ T cells network

Phenotypes of CD4+ T lymphocytes are highly plastic and adaptable to different environmental signals. This occurs to fine-tune immune response against to specific threatens. About phenotypes of CD4+ T cells, they can be classified according to their gene expression patterns. For instance, the immune response against virus, intracellular bacteria and cancer is mediated by Th1 cells, which signature is the presence of T-bet and IFN-g(121). On the other hand, Th2 phenotype is specialized to fight against parasites and allergens, and its signature is the presence of GATA3, as well as IL-2 and IL-4(122). Th9 phenotype is specialized against tumors and parasites, and its signature is the presence of transcription factor PU.1 and IL-9(12). Th17 phenotype is specialized in fighting against extracellular threatens such as fungi and bacteria, and its signature are transcription factor RORgT as well as IL-17 and IL-6(121). Finally, regulatory phenotypes are in charge to downregulate inflammation and may counteract specific response of effector phenotypes. In general terms, regulatory phenotypes may present the expression of FoxP3, IL-10 and TGF-b(123). Concerning genetic markers of Th1 phenotype, it has been observed that T-bet expression is promoted by IFN-g(69), IL-12(71) and by itself(124). On the other hand, GATA3(125), IL-4(126), and IL-6(127) might abrogate T-bet expression. In other words:

$$Tbet\left( t+1 \right)=\left( IFN\gamma\left( t \right)\vee\left( IL12\left( t \right)\wedge\neg\left( IL6\left( t \right)\vee IL4\left( t \right)\vee IL10\left( t \right) \right) \right) \right)\vee\left( Tbet\left( t \right) \right)\wedge\neg\left( IL6\left( t \right)\vee IL4\left( t \right)\vee GATA3\left( t \right) \right)$$

Concerning to IFN-g, this protein is able to induce its own expression(65), as well as T-bet does(65). On the contrary, the presence of GATA3 inhibits IFN-g expression(77), the same is true for TGF-b(63), that is:

$$IFN\gamma\left( t+1 \right)=\left( IFN\gamma^{*}\vee\left( \left( IFN\gamma\left( t \right)\vee Tbet\left( t \right) \right)\wedge\neg\left( GATA3\left( t \right)\vee TGF\beta\left( t \right) \right) \right) \right)\wedge\neg\left( IL4\left( t \right)\vee IL6\left( t \right)\vee IL10\left( t \right)\vee Ceramide^{*} \right)$$

Regarding Th2 markers, GATA3 expression can be enhanced by itself(128), as well as by IL-2 and IL-4(105). The contrary effect is obtained when there are high levels of T-bet(77), TGF-b(112), IFN-g (129), IL-6(11) and PU.1(130). This means that:

$$GATA3\left( t+1 \right)=\left( (IL2\left( t \right)\wedge IL4\left( t \right))\vee GATA3\left( t \right) \right)\wedge\neg\left( Tbet\left( t \right)\vee TGF\beta\left( t \right)\vee IFN\gamma\left( t \right)\vee IL6\left( t \right)\vee PU.1\left( t \right) \right)$$

The levels of IL-2 increase by its own activity(11), and are decreased by the presence of FoxP3(131), IL-6(132), IL-10(133) and IFN-g(134). In other words:

$$IL2\left( t+1 \right)=\left( IL2^{*}\vee\left( IL2\left( t \right)\wedge\neg FoxP3\left( t \right) \right) \right)\wedge\neg\left( IFN\gamma\left( t \right)\vee IL6\left( t \right)\vee\left( IL10\left( t \right)\wedge\neg FoxP3\left( t \right) \right) \right)$$

The levels of IL-4 increase by its own signaling activity(134), as well as by IL-2(134) and transcriptional functions of GATA3(105). On the other hand, they decrease when there are high levels of T-bet(106), IFN-g(126), IL-6(11) and PU.1(135), i.e.,

$$IL4\left( t+1 \right)=\left( IL4^{*}\vee\left( GATA3\left( t \right)\wedge\left( IL2\left( t \right)\vee IL4\left( t \right) \right) \right)\wedge\neg Tbet\left( t \right) \right)\wedge\neg\left( IFN\gamma\left( t \right)\vee IL6\left( t \right)\vee PU.1\left( t \right) \right)$$

On the other hand, a specific marker of Th17 phenotype is the transcription factor RORgT, which activation depends on the presence of TGF-b(136) and IL-6(137), and is downregulated by T-bet(138), GATA3(139), and FoxP3(140). In other words:

$$ROR\gamma T\left( t+1 \right)=\left( TGF\beta\left( t \right)\wedge IL6\left( t \right) \right)\wedge\neg\left( Tbet\left( t \right)\vee GATA3\left( t \right)\vee FoxP3\left( t \right) \right)$$

Concerning to IL-6, its expression is triggered by RORgT(11), and by its own signaling through STAT3 pathway(75). On the contrary, IL-6 is downregulated by IFN-g(75), IL-2, IL-4(11) and IL-10(141). Thus, this can be expressed as follows:

$$IL6\left( t+1 \right)=\left( ROR\gamma T\left( t \right)\vee IL6\left( t \right)\vee IL6^{*} \right)\wedge\neg\left( IFN\gamma\left( t \right)\vee IL2\left( t \right)\vee IL4\left( t \right)\vee IL10\left( t \right) \right)$$

In the same way, genetic markers of regulatory phenotypes can be controlled by several factors. In particular, the levels of FoxP3 can be increased by IL-2(142), ceramides(143), TGF-b(144) and by its own transcriptional activity(143). FoxP3 levels can be decreased by the presence of RORgT(11), IL-6(11), and PU.1(145). That is:

$$FoxP3\left( t+1 \right)=\left( IL2\left( t \right)\wedge\left( Ceramide^{*}\vee TGF\beta\left( t \right)\vee FoxP3\left( t \right) \right) \right)\wedge\neg\left( ROR\gamma T\left( t \right)\vee IL6\left( t \right)\vee PU.1\left( t \right) \right)$$

TGF-b levels can be augmented by its own signaling activity(114), as well as by FoxP3(11). The opposite effect is obtained in presence of high levels of IL-6(117). This implies that:

$$TGF\beta\left( t+1 \right)=TGF\beta^{*}\vee\left( TGF\beta\left( t \right)\vee FoxP3\left( t \right)\wedge\neg IL6\left( t \right) \right)$$

IL-10 levels can be busted by its own signaling activity through STAT3 activation(33,34), as well as by the presence of other cytokines such as IFN-g(146), TGF-b(11), IL-6 by STAT3 activation(75), IL-27(147) and by the transcriptional activity of GATA3(148). On the other hand, ceramides(149) and insulin(150) are able to abrogate the expression of IL-10. In other words:

$$IL10\left( t+1 \right)=IL{10}^{*}\vee\left( IL10\left( t \right)\wedge\left( IFN\gamma\left( t \right)\vee TGF\beta\left( t \right)\vee IL6\left( t \right)\vee IL{27}^{*}\vee GATA3\left( t \right) \right)\wedge\neg\left( Insulin\vee Ceramide^{*} \right) \right)$$

On the other hand, the levels of PU.1 can be increased by its own presence(135) as well as by the presence of TGF-b(151) and IL-6(152). PU.1 levels are decreased in presence of IL-2 through STAT5 activity(153), i.e.,

$$PU.1\left( t+1 \right)=\left( TGF\beta\left( t \right)\vee IL6\left( t \right)\vee PU.1\left( t \right) \right)\wedge\neg IL2\left( t \right)$$

Finally, IL-9 levels can be triggered by IL-4 as well as by high levels of PU.1(154), but they can be decreased as a result of T-bet activity(155), that is:

$$IL9\left( t+1 \right)=\left( \left( IL4^{*}\vee IL4\left( t \right) \right)\wedge PU.1\left( t \right) \right)\wedge\neg Tbet\left( t \right)$$

Collectively, these experimental data validate the quality of our reduced networks to represent the biology of adipocytes, macrophages and CD4+ T cells.

# Calculating fixed points for each network

We implemented each network model in Microsoft Visual Studio 2022 in a C# code. To calculate all attractors of each network we used a brute force-based approach, in which we first calculated the size of the space of states ($\Omega$) for each network, given by:

$$\Omega=2^{n}$$

Where “n” is the number of network nodes. Then, we calculated all possible initial configurations of the space of states for each network, a we use them to initiate a numerical simulation, in which we synchronously updated all nodes until we found fixed points ($p_{i}$), defined by the following property:

$$g\left( p_{i} \right)=p_{i}, \forall t$$

Where “g” is a set of Boolean equations that represent a specific network, and “$p_{i}$” is a fixed point or, in other words, a specific configuration of the space of states that indefinitely remains constant over time. Once the algorithm detects a fixed point, it counts how many times it appears from all initial configurations of the space of states of each network. With this approach, we were able to calculate fixed points and the size of the basin of attraction related to all of them at the same time. Our code for each network is available at(156).

Biologically, each fixed point represents a set of genes that are activated under certain circumstances, which is equivalent to the concept of “genotype”. In this sense, we used characteristic markers of all phenotypes presented in adipocytes, macrophages and CD4+ T cells to identify the corresponding phenotype of each genotype (i.e., genotype) found in this work. To do that, we used labelling rules for macrophages and CD4+ T cells, presented in Supplementary Tables 4 and 5, since these phenotypes are widely studied. On the other size, to identify phenotype of adipocytes, we proposed to define phenotypes for these cells based on their physiological state, which includes to know whether they are hypertrophic, or whether they are inflamed or whether they are insulin-responsive or not. To identify such characteristics, we used TNF as a marker of inflammation in adipocytes(46), CTGF as a marker of hypertrophy of adipocytes(46) and finally, we used the translocation of GLUT4 as a marker of insulin responsiveness of adipocytes(157), to obtain physiological phenotypes of these cells described in Supplementary Table 6. We show all fixed points for each network in Supplementary Figures 3, 4 and 5.

**Supplementary Table 4: Phenotypes of macrophages**

| **Phenotype** | **Logic labeling rule** |  |
| --- | --- | --- |
| $M0$ | $\neg iNOS\wedge\neg Arg1$ |  |
| $M1$ | $iNOS\wedge\neg Arg1$ |  |
| $M2$ | $\neg iNOS\wedge Arg1$ |  |
| $M1 TAM$ | $iNOS\wedge Arg1\wedge\left( IFN\gamma\vee IL12 \right)$ |  |
| $M2 TAM$ | $iNOS\wedge Arg1\wedge\neg\left( IFN\gamma\vee IL12 \right)$ |  |

**Supplementary Table 5:** **Phenotypes of CD4+ T cells**

| **Phenotype** | **Logic labeling rule** |  |
| --- | --- | --- |
| $Th0$ | $\neg\left( Tbet\vee GATA3\vee ROR\gamma T\vee FoxP3\vee TGF\beta\vee IL10\vee PU.1 \right)$ |  |
| $Th1$ | $\left( Tbet\wedge IFN\gamma\right)\wedge\neg(FoxP3\vee TGF\beta\vee IL10)$ |  |
| $Th2$ | $\left( GATA3\wedge(IL4\vee IL13) \right)\wedge\neg\left( FoxP3\vee TGF\beta\vee IL10 \right)$ |  |
| $Th9$ | $\left( PU.1\wedge IL9 \right)\wedge\neg\left( Tbet\vee IFN\gamma\vee GATA3\vee ROR\gamma T\vee IL4\vee FoxP3 \right)$ |  |
| $Th17$ | $ROR\gamma T\wedge(IL6\vee IL21)\wedge\neg IL10$ |  |
| $Th1R$ | $Tbet\wedge(FoxP3\vee TGF\beta\vee IL10)$ |  |
| $Th2R$ | $GATA3\wedge(FoxP3\vee TGF\beta\vee IL10)$ |  |
| $iTreg$ | $FoxP3\wedge TGF\beta\wedge\neg\left( Tbet\vee GATA3\vee ROR\gamma T \right)$ |  |
| $Tr1$ | $IL10\wedge\neg\left( FoxP3\vee GATA3\vee ROR\gamma T\vee TGF\beta\right)$ |  |
| $Th3$ | $TGF\beta\wedge\neg\left( FoxP3\vee GATA3\vee ROR\gamma T\vee Tbet \right)$ |  |

**Supplementary Table 6:** **Proposed phenotypes of adipocytes**

| **Phenotype** | **Logic labeling rule** |  |
| --- | --- | --- |
| No inflamed, no hypertrophic, no insulin responsive | $\neg TNF\wedge\neg CTGF \wedge\neg GLUT4$ |  |
| Inflamed, no hypertrophic, no insulin responsive | $TNF\wedge\neg CTGF \wedge\neg GLUT4$ |  |
| No inflamed, hypertrophic, no insulin responsive | $\neg TNF\wedge CTGF \wedge\neg GLUT4$ |  |
| Inflamed, hypertrophic, no insulin responsive | $TNF\wedge CTGF \wedge\neg GLUT4$ |  |
| No inflamed, no hypertrophic, insulin responsive | $\neg TNF\wedge\neg CTGF \wedge GLUT4$ |  |
| Inflamed, no hypertrophic, insulin responsive | $TNF\wedge\neg CTGF \wedge GLUT4$ |  |
| No inflamed, hypertrophic, insulin responsive | $\neg TNF\wedge CTGF \wedge GLUT4$ |  |
| Inflamed, hypertrophic, insulin responsive | $TNF\wedge CTGF \wedge GLUT4$ |  |


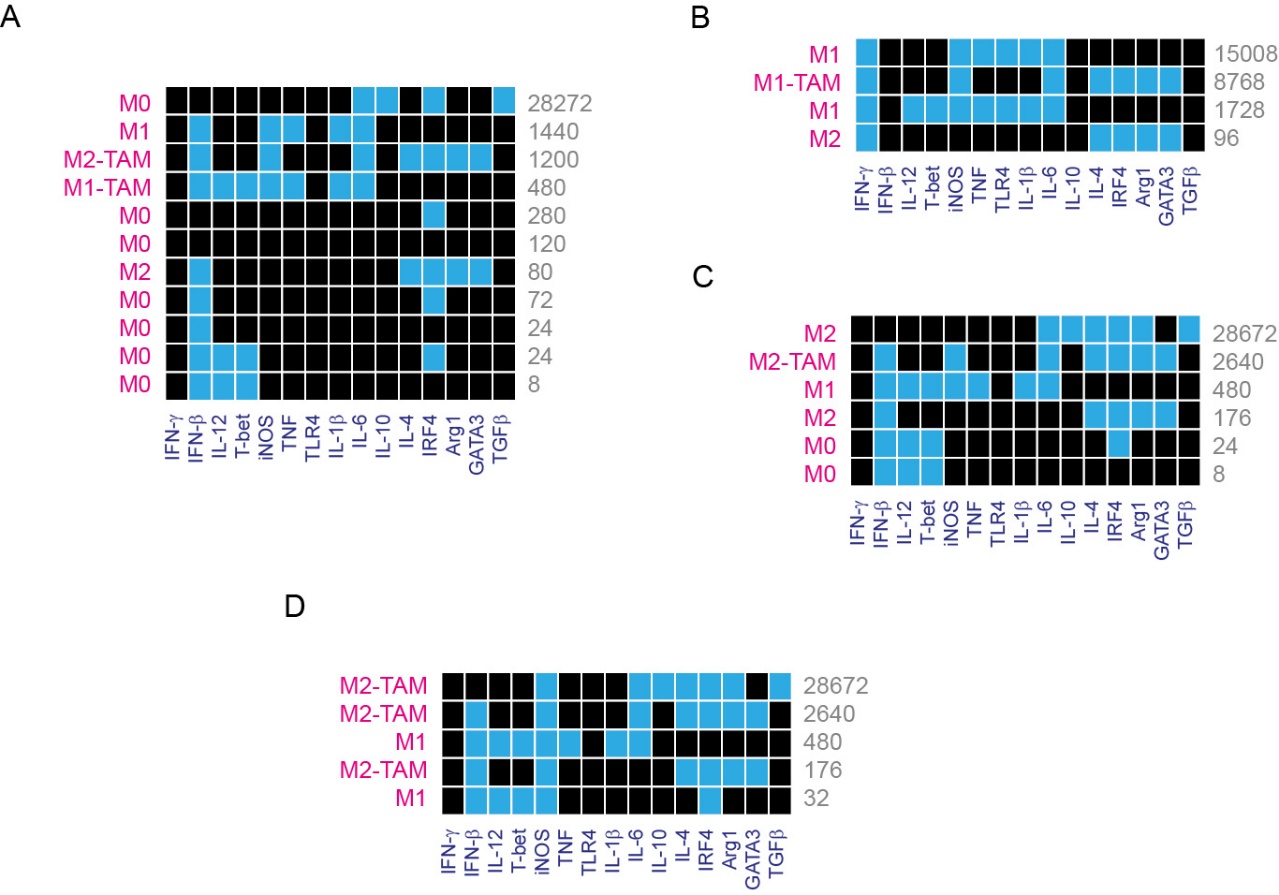


**Supplementary Figure 3: Attractors found for GRN of macrophages.** (A) Attractors found by under pro-M0 environment, that can be simulated by setting all inputs in zero. (B) Attractors found under pro-M1 environment, which is produced by external stimulation of TLR4 and IFN-g. (C) Attractors found under pro-M2 environment, which is simulated by setting “active” external IL-4 concentration. (D) Attractors found under pro-TAM environment, simulated by activating external IL-4, IL-10 and GM-CSF. Blue squares are used to indicate “active nodes” and black squares are used to mark “inactive nodes”. Blue labels show the name of each node of the macrophages network. Pink labels show the corresponding phenotype to each attractor and gray labels show the total count of initial states that converge to determined attractor. For this network, $\Omega= 32768$.


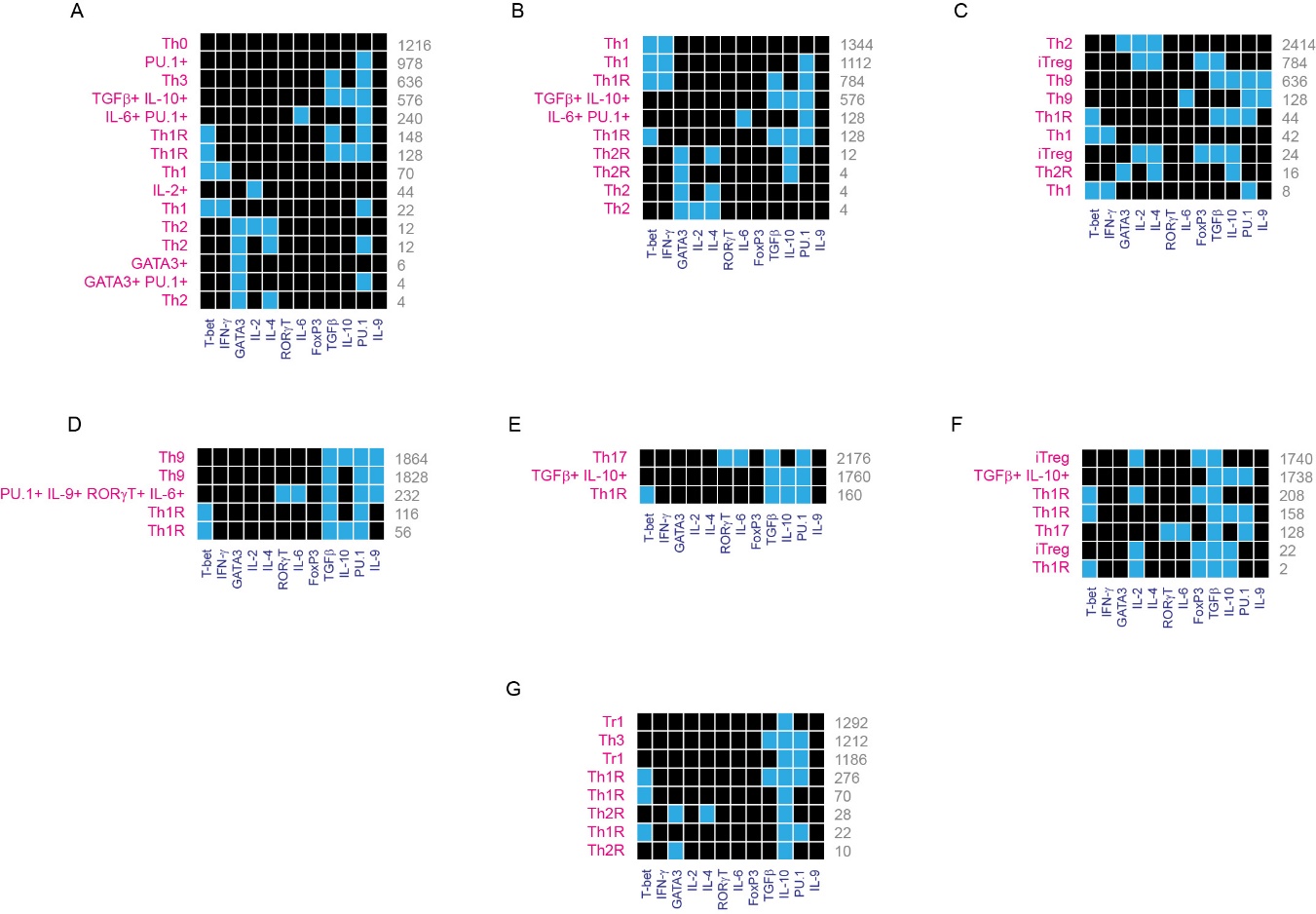


**Supplementary Figure 4: Attractors found for GRN of CD4+ T cells.** (A) Attractors found in pro-Th0 environment, simulated by setting “zero” in all inputs. (B) Attractors found in pro-Th1 environment, which is simulated by setting “one” in external IL-12 and IFN-g. (C) Attractors found in pro-Th2 environment, which is simulated by setting “one” in external IL-2 and IL-4. (D) Attractors found in pro-Th9 environment, simulated by setting “one” for external IL-4 and TGF-b. (E) Attractors found in pro-Th17 environment, simulated by setting “one” external IL-6 and TGF-b. (F) Attractors found in pro-iTreg environment, which is simulated by setting “one” in external IL-2 and TGF-b. (G) Attractors found in pro-Tr1 environment, simulated by setting “one” in external IL-10 and IL-27. Blue squares are used to indicate “active nodes” and black squares are used to mark “inactive nodes”. Blue labels show the name of each node of the CD4+ T cells network. Pink labels show the corresponding phenotype to each attractor and gray labels show the total count of initial states that converge to determined attractor. For this network, $\Omega= 4096$.


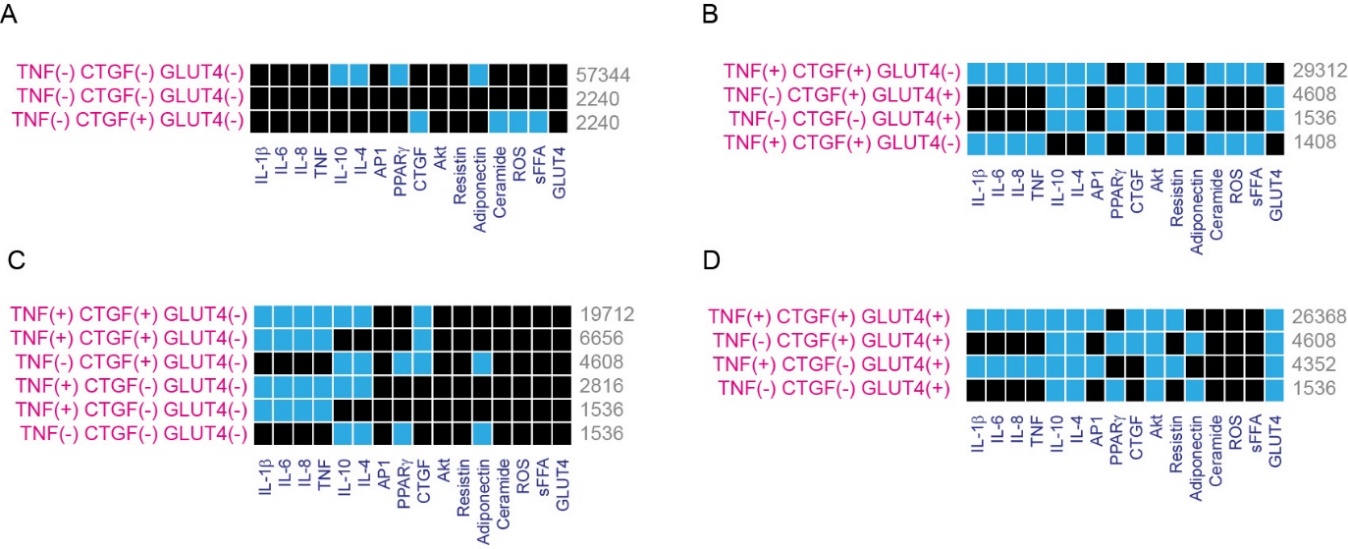


**Supplementary Figure 5: Attractors found for GRN of adipocytes.** (A) Attractors found in absence of stimuli, which is simulated by setting “zero” in all inputs. (B) Attractors found in obesity context, characterized by the presence of TNF, TNFR2, CTGF and insulin. This scenario was simulated by setting “one” in the above-mentioned inputs. (C) Attractors found in obesity without lipotoxicity before feeding, which is characterized by the presence of TNF, TNFR2, CTGF in absence of intracellular ceramides. This scenario was simulated by setting “one” in the above-mentioned inputs and by setting “zero” in the ceramide node. (D) Attractors found in obesity without lipotoxicity post-feeding, which is characterized by the presence of TNF, TNFR2, CTGF and insulin. Blue squares are used to indicate “active nodes” and black squares are used to mark “inactive nodes”. Blue labels show the name of each node of the adipocytes network. Pink labels show the corresponding phenotype to each attractor and gray labels show the total count of initial states that converge to determined attractor. For this network, $\Omega= 65536$.

# Details of stochastic modeling for each GRN

In order to construct each stochastic, we selected the most representative attractor for each cell phenotype. We assumed that when a specific attractor is reached by many initial conditions, then it should be the most probable form (i.e., genotype) that corresponds to specific phenotype. For instance, to represent the entire M0 phenotype, we selected the first attractor labelled as M0 of panel A at Supplementary Figure 3. We decided so, because this attractor was reached by 28272 initial configurations, compared to other attractors labelled as M0. We applied the same reasoning to other phenotypes for macrophages, CD4+ T cells and adipocytes. In Supplementary Figure 6, we show all attractors used to represent each phenotype of macrophages, CD4+ T cells and adipocytes.


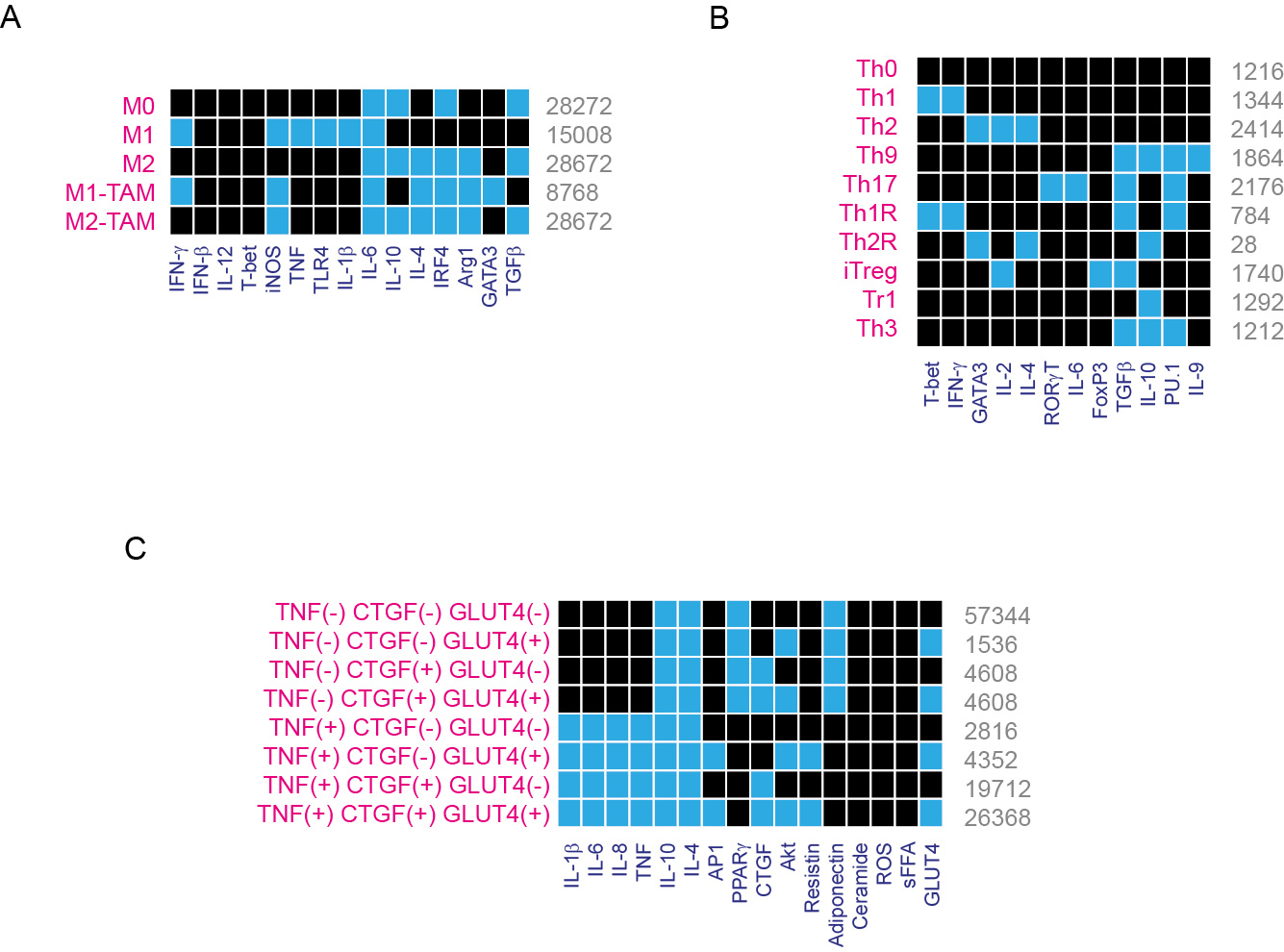


**Supplementary Figure 6: Attractors used to construct stochastic models.** (A) Attractors that represent all phenotypes of macrophages for this work. (B). Attractors that represent all phenotypes of CD4+ T cells studied in this work. (C) Attractors that represent all phenotypes of adipocytes analyzed in this work. Blue squares are used to indicate “active nodes” and black squares are used to mark “inactive nodes”. Blue labels show the name of each node of the adipocytes network. Pink labels show the corresponding phenotype to each attractor and gray labels show the total count of initial states that converge to determined attractor.

# Supplementary references

1. Acosta JR, Tavira B, Douagi I, Kulyté A, Arner P, Rydén M, et al. Human-Specific Function of IL-10 in Adipose Tissue Linked to Insulin Resistance. J Clin Endocrinol Metab. 2019;

2. Fang B, Chen X, Wu M, Kong H, Chu G, Zhou Z, et al. Luteolin inhibits angiogenesis of the M2-like TAMs via the downregulation of hypoxia inducible factor-1a and the STAT3 signalling pathway under hypoxia. Mol Med Rep. 2018;

3. Verboven K, Wouters K, Gaens K, Hansen D, Bijnen M, Wetzels S, et al. Abdominal subcutaneous and visceral adipocyte size, lipolysis and inflammation relate to insulin resistance in male obese humans. Sci Rep. 2018;

4. Aquilano K, Ceci V, Gismondi A, De Stefano S, Iacovelli F, Faraonio R, et al. Adipocyte metabolism is improved by TNF receptor-targeting small RNAs identified from dried nuts. Commun Biol. 2019;

5. Curiel TJ, Coukos G, Zou L, Alvarez X, Cheng P, Mottram P, et al. Specific recruitment of regulatory T cells in ovarian carcinoma fosters immune privilege and predicts reduced survival. Nat Med. 2004;

6. Makki K, Froguel P, Wolowczuk I. Adipose Tissue in Obesity-Related Inflammation and Insulin Resistance: Cells, Cytokines, and Chemokines. ISRN Inflamm. 2013;

7. Uhlén M, Fagerberg L, Hallström BM, Lindskog C, Oksvold P, Mardinoglu A, et al. Tissue-based map of the human proteome. Science (80- ). 2015;347(6220).

8. Kanehisa M, Goto S. KEGG: Kyoto Encyclopedia of Genes and Genomes. Nucleic Acids Research. 2000. p. 27–30.

9. Kanehisa M. Toward understanding the origin and evolution of cellular organisms. Protein Science. 2019. p. 1947–51.

10. Kanehisa M, Furumichi M, Sato Y, Ishiguro-Watanabe M, Tanabe M. KEGG: Integrating viruses and cellular organisms. Nucleic Acids Res. 2021;49(D1):D545–51.

11. Martinez-Sanchez ME, Hiriart M, Alvarez-Buylla ER. The CD4+ T cell regulatory network mediates inflammatory responses during acute hyperinsulinemia: A simulation study. BMC Syst Biol. 2017;11(1):64.

12. Jiang Y, Chen J, Bi E, Zhao Y, Qin T, Wang Y, et al. TNF-α enhances Th9 cell differentiation and antitumor immunity via TNFR2-dependent pathways. J Immunother Cancer. 2019;

13. Villarreal C, Padilla-Longoria P, Alvarez-Buylla ER. General theory of genotype to phenotype mapping: Derivation of epigenetic landscapes from n-node complex gene regulatory networks. Phys Rev Lett. 2012 Sep 12;109(11):118102.

14. Fain JN. Release of Interleukins and Other Inflammatory Cytokines by Human Adipose Tissue Is Enhanced in Obesity and Primarily due to the Nonfat Cells. Vol. 74, Vitamins and Hormones. 2006.

15. Rotter V, Nagaev I, Smith U. Interleukin-6 (IL-6) Induces Insulin Resistance in 3T3-L1 Adipocytes and Is, Like IL-8 and Tumor Necrosis Factor-α, Overexpressed in Human Fat Cells from Insulin-resistant Subjects. J Biol Chem. 2003;

16. Kobashi C, Asamizu S, Ishiki M, Iwata M, Usui I, Yamazaki K, et al. Inhibitory effect of IL-8 on insulin action in human adipocytes via MAP kinase pathway. J Inflamm. 2009;6.

17. Kucuksayan H H, Akgun S S. Pl3K/Akt/NF-κB Signalling Pathway on NSCLC Invasion. Med Chem (Los Angeles). 2016;06(04).

18. Burke SJ, Goff MR, Updegraff BL, Lu D, Brown PL, Minkin SC, et al. Regulation of the CCL2 Gene in Pancreatic β-Cells by IL-1β and Glucocorticoids: Role of MKP-1. PLoS One. 2012;7(10).

19. Álvarez S, Muñoz-Fernández MÁ. TNF-Α May Mediate Inflammasome Activation in the Absence of Bacterial Infection in More than One Way. PLoS One. 2013;8(8).

20. Chen WC, Lu YC, Kuo SJ, Lin CY, Tsai CH, Liu SC, et al. Resistin enhances IL-1β and TNF-α expression in human osteoarthritis synovial fibroblasts by inhibiting miR-149 expression via the MEK and ERK pathways. FASEB J. 2020;34(10).

21. Mukohda M, Stump M, Ketsawatsomkron P, Hu C, Quelle FW, Sigmund CD. Endothelial PPAR-γ provides vascular protection from IL-1β–induced oxidative stress. Am J Physiol - Hear Circ Physiol. 2016;310(1).

22. Bai D, Ueno L, Vogt PK. Akt-mediated regulation of NFκB and the essentialness of NFκB for the oncogenicity of PI3K and Akt. Int J Cancer. 2009;125(12).

23. Zhou A, Scoggin S, Gaynor RB, Williams NS. Identification of NF-kappa B-regulated genes induced by TNFalpha utilizing expression profiling and RNA interference. Oncogene [Internet]. 2003 Apr 3 [cited 2016 Jan 27];22(13):2054–64. Available from: http://www.ncbi.nlm.nih.gov/pubmed/12673210

24. Silswal N, Singh AK, Aruna B, Mukhopadhyay S, Ghosh S, Ehtesham NZ. Human resistin stimulates the pro-inflammatory cytokines TNF-α and IL-12 in macrophages by NF-κB-dependent pathway. Biochem Biophys Res Commun. 2005;334(4).

25. Brasier AR. The nuclear factor-kB-interleukin-6 signalling pathway mediating vascular inflammation. Available from: https://academic.oup.com/cardiovascres/article/86/2/211/374118

26. Whitham M, Pal M, Petzold T, Hjorth M, Egan CL, Brunner JS, et al. Adipocyte-specific deletion of IL-6 does not attenuate obesity-induced weight gain or glucose intolerance in mice. Am J Physiol - Endocrinol Metab. 2019;317(4).

27. Peng Y, Liu H, Liu F, Wang H, Liu Y, Duan S. Inhibitory effect of PPAR-Gamma activator on IL-6 and mPGES protein expression in PBMC induced by homocysteine. Hemodial Int. 2005;9(SUPPL. 1).

28. Elliott CL, Allport VC, Loudon JAZ, Wu GD, Bennett PR. Nuclear factor-kappa B is essential for up-regulation of interleukin-8 expression in human amnion and cervical epithelial cells. Vol. 7, Molecular Human Reproduction. 2001.

29. Adamiec R, Gacka M, Dobosz T, Szymaniec S, Bednarska-Chabowska D, Sadakierska-Chudy A. Stimulation of the peroxisome proliferator-activated receptor γ (PPARγ) and the expression of selected blood monocyte cytokine genes in diabetic macroangiopathy. Atherosclerosis. 2007 Oct 1;194(2):e108–15.

30. Stephens JM, Lee J, Pilch PF. Tumor necrosis factor-α-induced insulin resistance in 3T3-L1 adipocytes is accompanied by a loss of insulin receptor substrate-1 and GLUT4 expression without a loss of insulin receptor-mediated signal transduction. J Biol Chem. 1997;

31. Hayden MS, Ghosh S. Regulation of NF-κB by TNF Family Cytokines. 2014;

32. Choi EW, Lee M, Song JW, Kim K, Lee J, Yang J, et al. Fas mutation reduces obesity by increasing IL-4 and IL-10 expression and promoting white adipose tissue browning. Sci Rep. 2020;

33. Hutchins AP, Diez D, Miranda-Saavedra D. The IL-10/STAT3-mediated anti-inflammatory response: Recent developments and future challenges. Brief Funct Genomics. 2013;

34. Hedrich CM, Rauen T, Apostolidis SA, Grammatikos AP, Rodriguez NR, Ioannidis C, et al. Stat3 promotes IL-10 expression in lupus T cells through trans-activation and chromatin remodeling. Proc Natl Acad Sci U S A. 2014;

35. Mitchell RE, Hassan M, Burton BR, Britton G, Hill E V, Verhagen J, et al. IL-4 enhances IL-10 production in Th1 cells: implications for Th1 and Th2 regulation. Available from: www.nature.com/scientificreports/

36. Cao S, Zhang X, Edwards JP, Mosser DM. NF-κB1 (p50) homodimers differentially regulate pro- and anti-inflammatory cytokines in macrophages. J Biol Chem. 2006;281(36).

37. Silva-Filho JL, Caruso-Neves C, Pinheiro AAS. IL-4: an important cytokine in determining the fate of T cells.

38. Deimel LP, Li Z, Roy S, Ranasinghe C. STAT3 determines IL-4 signalling outcomes in naïve T cells. Sci Reports | [Internet]. 123AD;11:10495. Available from: https://doi.org/10.1038/s41598-021-89860-7

39. Ayala JE, Boustead JN, Chapman SC, Svitek CA, Oeser JK, Robey RB, et al. Insulin-mediated activation of activator protein-1 through the mitogen-activated protein kinase pathway stimulates collagenase-1 gene transcription in the MES 13 mesangial cell line. Vol. 33, Journal of Molecular Endocrinology. 2004.

40. Wang P, Anderson PO, Chen S, Paulsson KM, Sjögren HO, Li S. Inhibition of the transcription factors AP-1 and NF-κB in CD4 T cells by peroxisome proliferator-activated receptor γ ligands. Int Immunopharmacol. 2001 Apr 1;1(4):803–12.

41. Huang JT, Welch JS, Ricote M, Binder CJ, Willson TM, Kelly C, et al. Interleukin-4-dependent production of PPAR-γ ligands in macrophages by 12/15-lipoxygenase. Nat 1999 4006742. 1999 Jul 22;400(6742):378–82.

42. So RK, Kyung SL, Hee SP, Seoung JP, Kyung HM, Sun MJ, et al. Involvement of IL-10 in Peroxisome Proliferator-Activated Receptor γ-Mediated Anti-Inflammatory Response in Asthma. Mol Pharmacol. 2005 Dec 1;68(6):1568–75.

43. Wang Y, Wang H, Hegde V, Dubuisson O, Gao Z, Dhurandhar N V., et al. Interplay of pro- and anti-inflammatory cytokines to determine lipid accretion in adipocytes. Int J Obes 2013 3711. 2013 Feb 5;37(11):1490–8.

44. Merlin D, Sitaraman S V, Wang L, Walia B, Evans J, Gewirtz AT. IL-6 Induces NF-kB Activation in the Intestinal Epithelia. J Immunol Ref. 2019;171:3194–201.

45. Waite KJ, Floyd ZE, Arbour-Reily P, Stephens JM. Interferon-γ-induced Regulation of Peroxisome Proliferator-activated Receptor γ and STATs in Adipocytes *. J Biol Chem. 2001 Mar 9;276(10):7062–8.

46. Henninger AMJ, Eliasson B, Jenndahl LE, Hammarstedt A. Adipocyte hypertrophy, inflammation and fibrosis characterize subcutaneous adipose tissue of healthy, non-obese subjects predisposed to type 2 diabetes. PLoS One. 2014;

47. Balah A, Ezzat O, Akool ES. Vitamin E inhibits cyclosporin A-induced CTGF and TIMP-1 expression by repressing ROS-mediated activation of TGF-β/Smad signaling pathway in rat liver. Int Immunopharmacol. 2018 Dec 1;65:493–502.

48. Porstmann T, Griffiths B, Chung Y-L, Delpuech O, Griffiths JR, Downward J, et al. PKB/Akt induces transcription of enzymes involved in cholesterol and fatty acid biosynthesis via activation of SREBP. Oncogene [Internet]. 2005 Sep 29 [cited 2016 Jan 21];24(43):6465–81. Available from: http://dx.doi.org/10.1038/sj.onc.1208802

49. Sokolowska E, Blachnio-Zabielska A. The Role of Ceramides in Insulin Resistance. Frontiers in Endocrinology. 2019. p. e6.

50. Procaccini C, De Rosa V, Galgani M, Carbone F, La Rocca C, Formisano L, et al. Role of adipokines signaling in the modulation of T cells function. Front Immunol [Internet]. 2013 Jan 18 [cited 2016 Feb 2];4:332. Available from: http://journal.frontiersin.org/article/10.3389/fimmu.2013.00332/abstract

51. Carter S, Miard S, Roy-Bellavance C, Boivin L, Li Z, Pibarot P, et al. Sirt1 Inhibits Resistin Expression in Aortic Stenosis. PLoS One. 2012 Apr 6;7(4):e35110.

52. Shyu KG, Chua SK, Wang BW, Kuan P. Mechanism of inhibitory effect of atorvastatin on resistin expression induced by tumor necrosis factor- in macrophages. J Biomed Sci. 2009 May 27;16(1):1–12.

53. Patel L, Buckels AC, Kinghorn IJ, Murdock PR, Holbrook JD, Plumpton C, et al. Resistin is expressed in human macrophages and directly regulated by PPARγ activators. Biochem Biophys Res Commun. 2003 Jan 10;300(2):472–6.

54. Maeda N, Takahashi M, Funahashi T, Kihara S, Nishizawa H, Kishida K, et al. PPARγ Ligands Increase Expression and Plasma Concentrations of Adiponectin, an Adipose-Derived Protein. Diabetes. 2001 Sep 1;50(9):2094–9.

55. Chang E, Choi JM, Kim WJ, Rhee EJ, Oh KW, Lee WY, et al. Restoration of adiponectin expression via the ERK pathway in TNFα-treated 3T3-L1 adipocytes. Mol Med Rep. 2014 Aug 1;10(2):905–10.

56. Li X, Zhang D, Vatner DF, Goedeke L, Hirabara SM, Zhang Y, et al. Mechanisms by which adiponectin reverses high fat diet-induced insulin resistance in mice. Proc Natl Acad Sci U S A. 2020;

57. Li P-L, Zhang Y. Cross Talk Between Ceramide and Redox Signaling: Implications for Endothelial Dysfunction and Renal Disease.

58. Wu T, Trayhurn P, Roh H, Hartig SM, Masschelin PM, Cox AR, et al. The Impact of Oxidative Stress on Adipose Tissue Energy Balance. 2020;

59. Lee KS, Kim SR, Park SJ, Park HS, Min KH, Jin SM, et al. Peroxisome proliferator activated receptor-γ modulates reactive oxygen species generation and activation of nuclear factor-κB and hypoxia-inducible factor 1α in allergic airway disease of mice. J Allergy Clin Immunol. 2006 Jul 1;118(1):120–7.

60. Galadari S, Rahman A, Pallichankandy S, Galadari A, Thayyullathil F. Role of ceramide in diabetes mellitus: Evidence and mechanisms. Lipids Health Dis. 2013;

61. Armoni M, Harel C, Karnieli E. Transcriptional regulation of the GLUT4 gene: from PPAR-gamma and FOXO1 to FFA and inflammation. Trends Endocrinol Metab [Internet]. 2007 Apr [cited 2015 Dec 23];18(3):100–7. Available from: http://www.ncbi.nlm.nih.gov/pubmed/17317207

62. Jorgovanovic D, Song M, Wang L, Zhang Y. Roles of IFN-γin tumor progression and regression: A review. Biomarker Research. 2020.

63. Park IK, Letterio JJ, Gorham JD. TGF-β1 inhibition of IFN-γ-induced signaling and Th1 gene expression in CD4+ T cells is Smad3 independent but MAP kinase dependent. Mol Immunol. 2007;

64. Kumaran Satyanarayanan S, El Kebir D, Soboh S, Butenko S, Sekheri M, Saadi J, et al. IFN-β is a macrophage-derived effector cytokine facilitating the resolution of bacterial inflammation.

65. Hu X, Ivashkiv LB. Cross-regulation of Signaling and Immune Responses by IFN-γ and STAT1. Immunity. 2009 Oct 10;31(4):539.

66. Li W, Hofer MJ, Jung SR, Lim S-L, Campbell IL. IRF7-Dependent Type I Interferon Production Induces Lethal Immune-Mediated Disease in STAT1 Knockout Mice Infected with Lymphocytic Choriomeningitis Virus. J Virol. 2014 Jul;88(13):7578–88.

67. Sin WX, Li P, Yeong JPS, Chin KC. Activation and regulation of interferon-β in immune responses. Immunol Res 2012 531. 2012 Mar 13;53(1):25–40.

68. Grunwell JR, Yeligar SM, Stephenson S, Ping X Du, Gauthier TW, Fitzpatrick AM, et al. TGF-β1 Suppresses the Type I IFN Response and Induces Mitochondrial Dysfunction in Alveolar Macrophages. J Immunol. 2018 Mar 15;200(6):2115–28.

69. Lighvani AA, Frucht DM, Jankovic D, Yamane H, Aliberti J, Hissong BD, et al. T-bet is rapidly induced by interferon-γ in lymphoid and myeloid cells. Proc Natl Acad Sci U S A. 2001 Dec 18;98(26):15137–42.

70. Tsaous A, Hayes EM, Di Gregoli K, Bond AR, Bevan L, Thomas AC, et al. Plaque Size Is Decreased but M1 Macrophage Polarization and Rupture Related Metalloproteinase Expression Are Maintained after Deleting T-Bet in ApoE Null Mice. PLoS One. 2016 Feb 1;11(2):e0148873.

71. Verma ND, Hall BM, Plain KM, Robinson CM, Boyd R, Tran GT, et al. Interleukin-12 (IL-12p70) promotes induction of highly potent Th1-like CD4+CD25+ T regulatory cells that inhibit allograft rejection in unmodified recipients. Front Immunol. 2014;

72. Ylikoski E, Lund R, Kyläniemi M, Filén S, Kilpeläinen M, Savolainen J, et al. IL-12 up-regulates T-bet independently of IFN-γ in human CD4+ T cells. Eur J Immunol. 2005;

73. Seeliger C, Schyschka L, Kronbach Z, Wottge A, van Griensven M, Wildemann B, et al. Signaling pathway STAT1 is strongly activated by IFN-β in the pathogenesis of osteoporosis. 2015;

74. Iwata S, Mikami Y, Sun HW, Brooks SR, Jankovic D, Hirahara K, et al. The Transcription Factor T-bet Limits Amplification of Type I IFN Transcriptome and Circuitry in T Helper 1 Cells. Immunity. 2017 Jun 20;46(6):983-991.e4.

75. Costa-Pereira AP, Tininini S, Strobl B, Alonzi T, Schlaak JF, Is’harc H, et al. Mutational switch of an IL-6 response to an interferon-γ-like response. Proc Natl Acad Sci U S A. 2002 Jun 11;99(12):8043–7.

76. Afkarian M, Sedy JR, Yang J, Jacobson NG, Cereb N, Yang SY, et al. T-bet is a STAT1-induced regulator of IL-12R expression in naïve CD4+ T cells. Nat Immunol 2002 36. 2002 May 13;3(6):549–57.

77. Kanhere A, Hertweck A, Bhatia U, Gökmen MR, Perucha E, Jackson I, et al. T-bet and GATA3 orchestrate Th1 and Th2 differentiation through lineage-specific targeting of distal regulatory elements. Nat Commun 2012 31. 2012 Dec 11;3(1):1–12.

78. Park I-K, Shultz LD, Letterio JJ, Gorham JD. TGF-beta1 inhibits T-bet induction by IFN-gamma in murine CD4+ T cells through the protein tyrosine phosphatase Src homology region 2 domain-containing phosphatase-1. J Immunol. 2005 Nov 1;175(9):5666–74.

79. Lisi L, Ciotti GMP, Braun D, Kalinin S, Currò D, Dello Russo C, et al. Expression of iNOS, CD163 and ARG-1 taken as M1 and M2 markers of microglial polarization in human glioblastoma and the surrounding normal parenchyma. Neurosci Lett. 2017 Apr 3;645:106–12.

80. Jiang N, Tian Z, Tang J, Ou R, Xu Y. Granulocyte Macrophage-Colony Stimulating Factor (GM-CSF) Downregulates the Expression of Protumor Factors Cyclooxygenase-2 and Inducible Nitric Oxide Synthase in a GM-CSF Receptor-Independent Manner in Cervical Cancer Cells. Mediators Inflamm. 2015;2015.

81. Riquelme P, Tomiuk S, Kammler A, Fändrich F, Schlitt HJ, Geissler EK, et al. IFN-γ-induced iNOS expression in mouse regulatory macrophages prolongs allograft survival in fully immunocompetent recipients. Mol Ther. 2013 Feb 1;21(2):409–22.

82. Müller E, Speth M, Christopoulos PF, Lunde A, Avdagic A, Øynebråten I, et al. Both type I and type II interferons can activate antitumor M1 macrophages when combined with TLR stimulation. Front Immunol. 2018;

83. Fonseca SG, Romão PRT, Figueiredo F, Morais RH, Lima HC, Ferreira SH, et al. TNF-α  mediates the induction of nitric oxide synthase in macrophages but not in neutrophils in experimental cutaneous leishmaniasis. Eur J Immunol. 2003 Aug 1;33(8):2297–306.

84. Teng X, Zhang H, Snead C, Catravas AJD. Molecular mechanisms of iNOS induction by IL-1β and IFN-γ in rat aortic smooth muscle cells. Am J Physiol - Cell Physiol. 2002;282(1 51-1).

85. Díaz-Guerra MJM, Castrillo A, Martín-Sanz P, Boscá L. Negative regulation by protein tyrosine phosphatase of IFN-γ-dependent expression of inducible nitric oxide synthase. J Immunol. 1999;162(11).

86. Han HE, Kim TK, Son HJ, Park WJ, Han PL. Activation of autophagy pathway suppresses the expression of iNOS, IL6 and cell death of LPS-stimulated microglia cells. Biomol Ther. 2013;21(1).

87. Schütze S, Wiegmann K, Machleidt T, Krönke M. TNF-induced activation of NF-kappa B. Immunobiology. 1995;193(2–4):193–203.

88. Shakibaei M, John T, Schulze-Tanzil G, Lehmann I, Mobasheri A. Suppression of NF-κB activation by curcumin leads to inhibition of expression of cyclo-oxygenase-2 and matrix metalloproteinase-9 in human articular chondrocytes: Implications for the treatment of osteoarthritis. Biochem Pharmacol. 2007 May 1;73(9):1434–45.

89. Wang J, Ford HR, Grishin A V. NF-kappaB-mediated expression of MAPK phosphatase-1 is an early step in desensitization to TLR ligands in enterocytes. Mucosal Immunol [Internet]. 2010 Sep [cited 2016 Jan 27];3(5):523–34. Available from: http://www.ncbi.nlm.nih.gov/pubmed/20555314

90. Brodbeck WG, Shive MS, Colton E, Ziats NP, Anderson JM. Interleukin-4 inhibits tumor necrosis factor-α-induced and spontaneous apoptosis of biomaterial-adherent macrophages. J Lab Clin Med. 2002;139(2).

91. Shin DI, Banning U, Kim YM, Verheyen J, Hannen M, Bönig H, et al. Interleukin 10 inhibits TNF-alpha production in human monocytes independently of interleukin 12 and interleukin 1 beta. Immunol Invest. 1999;28(2–3).

92. Guijarro-Muñoz I, Compte M, Álvarez-Cienfuegos A, Álvarez-Vallina L, Sanz L. Lipopolysaccharide activates toll-like receptor 4 (TLR4)-mediated NF-κB signaling pathway and proinflammatory response in human pericytes. J Biol Chem. 2014;289(4).

93. Fischer H, Lutay N, Ragnarsdóttir B, Yadav M, Jönsson K, Urbano A, et al. Pathogen specific, IRF3-dependent signaling and innate resistance to human kidney infection. PLoS Pathog. 2010;6(9).

94. Bryant AH, Spencer-Harty S, Owens SE, Jones RH, Thornton CA. Interleukin 4 and interleukin 13 downregulate the lipopolysaccharide-mediated inflammatory response by human gestation-associated tissues. Biol Reprod. 2017;96(3).

95. Hashimoto K, Otero M, Imagawa K, De Andrés MC, Coico JM, Roach HI, et al. Regulated transcription of human matrix metalloproteinase 13 (MMP13) and interleukin-1β (IL1B) genes in chondrocytes depends on methylation of specific proximal promoter CpG sites. J Biol Chem. 2013;288(14).

96. te Velde A, Huijbens R, Heije K, de Vries J, Figdor C. Interleukin-4 (IL-4) inhibits secretion of IL-1 beta, tumor necrosis factor alpha, and IL-6 by human monocytes. Blood. 1990;76(7).

97. Sun Y, Ma J, Li D, Li P, Zhou X, Li Y, et al. Interleukin-10 inhibits interleukin-1β production and inflammasome activation of microglia in epileptic seizures. J Neuroinflammation. 2019;16(1).

98. Park JI, Lee MG, Cho K, Park BJ, Chae KS, Byun DS, et al. Transforming growth factor-beta1 activates interleukin-6 expression in prostate cancer cells through the synergistic collaboration of the Smad2, p38-NF-kappaB, JNK, and Ras signaling pathways. Urol Oncol Semin Orig Investig. 2004;22(1).

99. Martinez FO, Gordon S. The M1 and M2 paradigm of macrophage activation: Time for reassessment. F1000Prime Rep. 2014;

100. Yang M, Song L, Wang L, Yukht A, Ruther H, Li F, et al. Deficiency of GATA3-Positive Macrophages Improves Cardiac Function Following Myocardial Infarction or Pressure Overload Hypertrophy. J Am Coll Cardiol. 2018;72(8).

101. Cao Q, Wang Y, Zheng D, Sun Y, Wang Y, Lee VWS, et al. IL-10/TGF-β-modified macrophages induce regulatory T cells and protect against adriamycin nephrosis. J Am Soc Nephrol. 2010;

102. Sanin DE, Prendergast CT, Mountford AP. IL-10 Production in Macrophages Is Regulated by a TLR-Driven CREB-Mediated Mechanism That Is Linked to Genes Involved in Cell Metabolism. J Immunol. 2015;195(3).

103. Hu X, Paik PK, Chen J, Yarilina A, Kockeritz L, Lu TT, et al. IFN-γ Suppresses IL-10 Production and Synergizes with TLR2 by Regulating GSK3 and CREB/AP-1 Proteins. Immunity. 2006;24(5).

104. Feng X, Yau D, Holbrook C, Reder AT. Type I interferons inhibit interleukin-10 production in activated human monocytes and stimulate IL-10 in T cells: Implications for Th1-mediated diseases. J Interf Cytokine Res. 2002;22(3).

105. Li-Weber M, Krammer PH. Regulation of IL4 gene expression by T cells and therapeutic perspectives. Nature Reviews Immunology. 2003.

106. Zhuang Y, Huang Z, Nishida J, Brown M, Zhang L, Huang H. A continuous T-bet expression is required to silence the interleukin-4-producing potential in T helper type 1 cells. Immunology. 2009;

107. Huang SC-C, Smith AM, Everts B, Colonna M, Pearce EL, Schilling JD, et al. mTORC2-IRF4 mediated metabolic reprograming is essential for macrophage alternative activation. Immunity. 2016;45(4).

108. Boddicker RL, Kip NS, Xing X, Zeng Y, Yang ZZ, Lee JH, et al. The oncogenic transcription factor IRF4 is regulated by a novel CD30/NF-kB positive feedback loop in peripheral T-cell lymphoma. Blood. 2015;125(20).

109. Sahoo A, Wali S, Nurieva R. T helper 2 and T follicular helper cells: Regulation and function of interleukin-4. Vol. 30, Cytokine and Growth Factor Reviews. 2016.

110. Ngwa C, Mamun A Al, Xu Y, Sharmeen R, Liu F. Phosphorylation of microglial IRF5 and IRF4 by IRAK4 regulates inflammatory responses to ischemia. Cells. 2021;10(2).

111. Liu YC, Zou XB, Chai YF, Yao YM. Macrophage polarization in inflammatory diseases. Vol. 10, International Journal of Biological Sciences. 2014.

112. Gorelik L, Fields PE, Flavell RA. Cutting Edge: TGF-β Inhibits Th Type 2 Development Through Inhibition of GATA-3 Expression. J Immunol. 2000;165(9).

113. Jiang T, Qu JJ, Nishinaka T, Zhang N. Transcription factor AP-1 regulates TGF-β1-induced expression of aldose reductase in cultured human mesangial cells. Nephrology. 2008;13(3).

114. Kim S Il, Choi ME. TGF-β-activated kinase-1: New insights into the mechanism of TGF-β signaling and kidney disease. Vol. 31, Kidney Research and Clinical Practice. 2012.

115. Zhou X, Spittau B, Krieglstein K. TGFβ signalling plays an important role in IL4-induced alternative activation of microglia. J Neuroinflammation. 2012;9.

116. Bagheri Y, Babaha F, Falak R, Yazdani R, Azizi G, Sadri M, et al. IL-10 induces TGF-β secretion, TGF-β receptor II upregulation, and IgA secretion in B cells. Eur Cytokine Netw. 2019;30(3).

117. Luckett-Chastain LR, Gallucci RM. Interleukin (IL)-6 modulates transforming growth factor-b expression in skin and dermal fibroblasts from IL-6-deficient mice. Br J Dermatol. 2009;161(2).

118. Huang Y, Wang Y, Lin L, Wang P, Jiang L, Liu J, et al. Overexpression of miR-133a-3p inhibits fibrosis and proliferation of keloid fibroblasts by regulating IRF5 to inhibit the TGF-β/Smad2 pathway. Mol Cell Probes. 2020;52.

119. Liu ZW, Zhang YM, Zhang LY, Zhou T, Li YY, Zhou GC, et al. Duality of Interactions Between TGF-β and TNF-α During Tumor Formation. Vol. 12, Frontiers in Immunology. 2022.

120. Thompson CD, Matta B, Barnes BJ. Therapeutic targeting of IRFs: Pathway-dependence or structure-based? Vol. 9, Frontiers in Immunology. 2018.

121. Tay RE, Richardson EK, Toh HC. Revisiting the role of CD4+ T cells in cancer immunotherapy—new insights into old paradigms. Vol. 28, Cancer Gene Therapy. 2021.

122. Abebe F. Synergy between Th1 and Th2 responses during Mycobacterium tuberculosis infection: A review of current understanding. International Reviews of Immunology. 2019.

123. Peterson RA. Regulatory T-Cells: Diverse Phenotypes Integral to Immune Homeostasis and Suppression. Toxicol Pathol. 2012;40(2).

124. Zhu J, Jankovic D, Oler AJ, Wei G, Sharma S, Hu G, et al. The Transcription Factor T-bet Is Induced by Multiple Pathways and Prevents an Endogenous Th2 Cell Program during Th1 Cell Responses. Immunity. 2012;

125. Kaminuma O, Kitamura F, Kitamura N, Miyagishi M, Taira K, Yamamoto K, et al. GATA-3 suppresses IFN-γ promoter activity independently of binding to cis-regulatory elements. FEBS Lett. 2004;

126. Nekoua MP, Fachinan R, Atchamou AK, Nouatin O, Amoussou-Guenou D, Amoussou-Guenou MK, et al. Modulation of immune cells and Th1/Th2 cytokines in insulin-treated type 2 diabetes mellitus. Afr Health Sci. 2016;

127. Wu W, Dietze KK, Gibbert K, Lang KS, Trilling M, Yan H, et al. TLR ligand induced IL-6 counter-regulates the anti-viral CD8+ T cell response during an acute retrovirus infection. Sci Rep. 2015;5.

128. Wan YY. GATA3: A master of many trades in immune regulation. Trends in Immunology. 2014.

129. Gonzales-van Horn SR, Farrar JD. Interferon at the crossroads of allergy and viral infections. J Leukoc Biol. 2015;

130. Yashiro T, Kubo M, Ogawa H, Okumura K, Nishiyama C. PU.1 suppresses Th2 cytokine expression via silencing of GATA3 transcription in dendritic cells. PLoS One. 2015;10(9).

131. Popmihajlov Z, Smith KA. Negative feedback regulation of T cells via interleukin-2 and FOXP3 reciprocity. PLoS One. 2008;3(2).

132. Papillion A, Powell MD, Chisolm DA, Bachus H, Fuller MJ, Weinmann AS, et al. Inhibition of IL-2 responsiveness by IL-6 is required for the generation of GC-TFH cells. Sci Immunol. 2019;4(39).

133. Taga K, Tosato G. IL-10 inhibits human T cell proliferation and IL-2 production. J Immunol. 1992;148(4).

134. Goleva E, Li NB, Leung DYM. IFN-γ Reverses IL-2– and IL-4–Mediated T-Cell Steroid Resistance. Am J Respir Cell Mol Biol. 2009 Feb 1;40(2):223.

135. Chang HC, Sehra S, Goswami R, Yao W, Yu Q, Stritesky GL, et al. The transcription factor PU.1 is required for the development of IL-9-producing T cells and allergic inflammation. Nat Immunol. 2010;11(6).

136. Manel N, Unutmaz D, Littman DR. The differentiation of human TH-17 cells requires transforming growth factor-β and induction of the nuclear receptor RORγt. Nat Immunol. 2008;9(6).

137. Yamashita T, Iwakura T, Matsui K, Kawaguchi H, Obana M, Hayama A, et al. IL-6-mediated Th17 differentiation through RORγt is essential for the initiation of experimental autoimmune myocarditis. Cardiovasc Res. 2011;91(4).

138. Wang Y, Godec J, Ben-Aissa K, Cui K, Zhao K, Pucsek AB, et al. The transcription factors T-bet and runx are required for the ontogeny of pathogenic interferon-γ-producing T helper 17 cells. Immunity. 2014;40(3).

139. Zhong C, Cui K, Wilhelm C, Hu G, Mao K, Belkaid Y, et al. Group 3 innate lymphoid cells continuously require the transcription factor GATA-3 after commitment. Nat Immunol. 2016;17(2).

140. Ichiyama K, Yoshida H, Wakabayashi Y, Chinen T, Saeki K, Nakaya M, et al. Foxp3 Inhibits RORγt-mediated IL-17A mRNA Transcription through Direct Interaction with RORγt *♦. J Biol Chem. 2008 Jun 20;283(25):17003–8.

141. HEMPEL L, KÖRHOLZ D, BÖNIG H, SCHNEIDER M, KLEIN‐VEHNE A, PACKEISEN J, et al. Interleukin‐10 Directly Inhibits the Interleukin‐6 Production in T‐Cells. Scand J Immunol. 1995;41(5).

142. Zorn E, Nelson EA, Mohseni M, Porcheray F, Kim H, Litsa D, et al. IL-2 regulates FOXP3 expression in human CD4+CD25+ regulatory T cells through a STAT-dependent mechanism and induces the expansion of these cells in vivo. Blood. 2006;

143. Kue CS, Lim HX, Jung MY, Hong HJ, Cho D, Kim TS. C6-ceramide in combination with transforming growth factor-β enhances Treg cell differentiation and stable FoxP3 expression in vitro and in vivo. Immunobiology. 2013;218(7).

144. Zhou L, Lopes JE, Chong MMW, Ivanov II, Min R, Victora GD, et al. TGF-Β-induced Foxp3 inhibits TH17 cell differentiation by antagonizing RORγt function. Nature. 2008;453(7192).

145. Maruyama T, Konkel JE, Zamarron BF, Chen WJ. The molecular mechanisms of Foxp3 gene regulation. Vol. 23, Seminars in Immunology. 2011.

146. Liu XS, Leerberg J, MacDonald K, Leggatt GR, Frazer IH. IFN-γ Promotes Generation of IL-10 Secreting CD4 + T Cells that Suppress Generation of CD8 Responses in an Antigen-Experienced Host . J Immunol. 2009;183(1).

147. Wehrens EJ, Wong KA, Gupta A, Khan A, Benedict CA, Zuniga EI. IL-27 regulates the number, function and cytotoxic program of antiviral CD4 T cells and promotes cytomegalovirus persistence. PLoS One. 2018;13(7).

148. Shoemaker J, Saraiva M, O’Garra A. GATA-3 Directly Remodels the IL-10 Locus Independently of IL-4 in CD4 + T Cells . J Immunol. 2006;

149. Chiba N, Masuda A, Yoshikai Y, Matsuguchi T. Ceramide inhibits LPS-induced production of IL-5, IL-10, and IL-13 from mast cells. J Cell Physiol. 2007;213(1).

150. Han JM, Patterson SJ, Speck M, Ehses JA, Levings MK. Insulin Inhibits IL-10–Mediated Regulatory T Cell Function: Implications for Obesity. J Immunol. 2014;192(2).

151. Travis MA, Sheppard D. TGF-β activation and function in immunity. Vol. 32, Annual Review of Immunology. 2014.

152. Tissères P, Araud T, Ochoda A, Drifte G, Dunn-Siegrist I, Pugin J. Cooperation between PU.1 and CAAT/enhancer-binding protein β is necessary to induce the expression of the MD-2 gene. J Biol Chem. 2009;284(39).

153. van de Laar L, van den Bosch A, Wierenga ATJ, Janssen HLA, Coffer PJ, Woltman AM. Tight Control of STAT5 Activity Determines Human CD34-Derived Interstitial Dendritic Cell and Langerhans Cell Development. J Immunol. 2011;186(12).

154. Goswami R, Kaplan MH. A Brief History of IL-9. J Immunol. 2011;186(6).

155. Zhang Y, Siegel AM, Sun G, Dimaggio T, Freeman AF, Milner JD. Human TH9 differentiation is dependent on signal transducer and activator of transcription (STAT) 3 to restrain STAT1-mediated inhibition. J Allergy Clin Immunol. 2019 Mar 1;143(3):1108-1118.e4.

156. Bensussen A. Boolean model of visceral adipose tissue. 2022 Jun 27 [cited 2022 Jun 27]; Available from: https://doi.org/10.5281/zenodo.6762235#.YroUOfsSi1Q.mendeley

157. Carruthers NJ, Strieder-Barboza C, Caruso JA, Flesher CG, Baker NA, Kerk SA, et al. The human type 2 diabetes-specific visceral adipose tissue proteome and transcriptome in obesity. Sci Rep. 2021;
